# What Is Regulating Chironomid Populations? The Influence of Food Supply and Interference Competition on Development and Mortality in *Chironomus riparius*

**DOI:** 10.1002/ece3.71949

**Published:** 2025-08-13

**Authors:** Tido Strauss, Jana Gerhard, Daniel Gerth, Josef Koch, Richard Ottermanns, Maxime Vaugeois, Nika Galic

**Affiliations:** ^1^ gaiac Research Institute Aachen Germany; ^2^ Institute for Environmental Research, RWTH Aachen University Aachen Germany; ^3^ Syngenta Crop Protection LLC Greensboro North Carolina USA; ^4^ Syngenta Crop Protection AG Basel Switzerland

**Keywords:** cannibalism, density dependence, Diptera, dynamic energy budget model DEB, individual‐based model IBM, interference competition

## Abstract

Density‐dependent processes are important for a fundamental understanding of population regulation, as well as for understanding responses to and recovery from stressors. While exploitative competition is well‐studied, interference competition is rather difficult to investigate, but it has been regularly observed to occur in many aquatic insect populations. We conducted laboratory experiments with the non‐biting midge *Chironomus riparius* (Diptera: Chironomidae) to investigate the impact of different combinations of food supply and larval densities on development and mortality at a constant temperature of 20°C. The chosen two‐factorial experimental design allowed a separate evaluation of exploitative (food) and interference (mortality) competition across a gradient of larval densities. The use of different vessel sizes between 50 cm^2^ and 600 cm^2^ made it possible to quantify the functional response at different food densities. To test mechanistic explanations for the statistically significant empirical relationships found in this study and to predict density‐dependent processes, we used a dynamic process‐oriented modeling approach. We extended a recently developed DEB‐IBM full life cycle model for *C. riparius* and successfully applied it under variable food conditions at the population level under laboratory conditions. Our study showed that chironomid development and reproduction are primarily dependent on food supply, whereas larval density drives the density‐dependent mortality rate. The interaction of food availability and interference competition determined the effective mortality over time. Killing by conspecifics was the most likely mechanism responsible for the intraspecific mortality of the larval stages. Combining data generated using a tailor‐made experimental design with a mechanistic model provided insights into and quantified regulation mechanisms of chironomid populations, allowing future uses of this information in the context of population‐level risk assessment from exposure to chemicals.

## Graphical abstract

Density‐dependent processes are important for a fundamental understanding of the regulation of populations. Laboratory experiments were conducted with the non‐biting midge *Chironomus riparius* (Diptera: Chironomidae) to investigate the impact of different combinations of food supply and larval densities on development and mortality. Our study showed that chironomid development and reproduction are primarily dependent on food, whereas larval density drives the density‐dependent mortality rate, with cannibalism being the most likely mechanism responsible for the intraspecific mortality of larval stages. The effective larval mortality over time for *C. riparius* under laboratory conditions is ultimately determined by the interaction of food availability and interference competition.

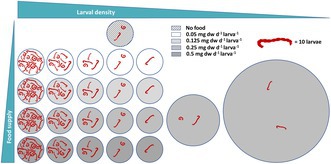

## 1 Introduction

Density‐dependent mechanisms are key to the regulation of stable, persistent animal populations (Sinclair 1989; Hooper, Sibly, Maund, and Hutchinson 2003; Elliott 2013; Accolla et al. 2023), and their functional understanding is essential for population prognosis and management (Hixon et al. 2002). In this context, density‐dependent mechanisms must be distinguished from density‐independent environmental factors (e.g., weather conditions and predation), catastrophic environmental conditions, and exposure to toxic pollutants, which can also limit populations regardless of their density (McIntosh et al. 2022; Accolla et al. 2023).

While both positive and negative density dependence exist in nature, we here focus on the negative density dependence in the regulation of populations, in which populations are reduced at high densities (Hixon et al. 2002; McIntosh et al. 2022; Accolla et al. 2023). Competition is an example of a negative density‐dependent process and can occur either indirectly as exploitative competition, where individuals compete for a limiting resource, such as food or space, or as interference competition directly between individuals, often via aggressive behavior (Le Bourlot et al. 2014; Accolla et al. 2023). In its extreme form, interference competition can result in the death of one of the individuals involved through injury or even direct consumption by the competitor (predation or cannibalism) (Fox 1975; Sokame et al. 2023). Cannibalism is considered a prime example of an intraspecific, density‐dependent mortality factor (Rosenheim and Schreiber 2022) and is one of the major mortality factors in many populations (Polis 1981). Cannibalism‐induced mortality can thus contribute to the self‐regulation of population densities by dampening oscillations and stabilizing fluctuations (Polis 1981; Claessen et al. 2004; Strauss et al. 2016; Rosenheim and Schreiber 2022; Accolla et al. 2023).

Of forty‐seven insect case studies examined by Sinclair (1989), most reported density‐dependent mortality at the larval stage, while density‐dependent reduction in fecundity or egg production was the second most common. More recent field studies with aquatic insects show that density‐dependent mortality stabilizes the population density of aquatic macroinvertebrates (Hildrew et al. 2004; Elliott 2013; Marchant 2021; McIntosh et al. 2022) and that combinations of exploitation and interference competition (including cannibalism), as well as density‐independent factors, are frequently observed under outdoor conditions (Hildrew et al. 2004; Marchant 2021).

In populations of chironomids, as in many other heterotrophic species, food quality and quantity have been shown to have a direct influence on their growth and development (Tokeshi 1995; Ristola et al. 1999; Péry et al. 2002). Several studies demonstrated that density‐dependent intraspecific competition affects chironomid larval growth mainly through the exploitation of food resources (Rasmussen 1985; Tokeshi 1995). The interpretation of the seasonal dynamics of chironomid populations is subject to considerable uncertainty, with the density‐dependent mortality of newly hatched larvae until emergence as adults playing an important role (Tokeshi 1995). However, current understanding of density‐dependent mortality in chironomids is still lacking; yet, it is crucial for assessing risks posed to field populations by stress factors, and should therefore be incorporated in population models (Accolla et al. 2023).

To fill this knowledge gap, in this study, we investigated intraspecific density‐dependent mortality in chironomids, considering exploitation and interference competition separately. To this end, we first designed tailor‐made experiments to generate relevant development and mortality data under several feeding and density conditions. Data were first analyzed statistically; followed by mechanistic modeling. Mechanistic models, here specifically population models, are valuable tools to test hypotheses about mechanisms of density dependence, improving our understanding of how these mechanisms influence population dynamics and can be extended to predict dynamics at broader scales (Strauss et al. 2016; Accolla et al. 2023).

To gain insight into and quantify the role of food supply and larval density on population regulation mechanisms in aquatic invertebrates, laboratory tests were performed with *Chironomus riparius* (Meigen, 1804; Syn.: *Chironomus thummi* Kieffer, 1911), a non‐biting midge (Diptera: Chironomidae) that is widely distributed in lotic and lentic waters in the northern hemisphere (Péry et al. 2002) and is a common test organism in ecotoxicological studies for environmental risk assessment (e.g., OECD 2023). Its life cycle includes aquatic stages (egg, four benthic larval instars, and a pupal stage) and, after emergence, an aerial adult stage (Prata et al. 2023). *C. riparius* is considered a sediment and deposit‐feeding midge (detritus, bacteria, periphyton, Pillot 2014; Prata et al. 2023), which feeds on the sediment surface (Naylor and Rodrigues 1995; Åkerblom and Goedkoop 2003; Prata et al. 2023) and is only exceptionally predatory (e.g., on small tubificids, Pillot 2014).

### 1.1 Study‐Specific Remarks

Commonly, laboratory experiments with *C. riparius* are conducted with a variable daily diet per larva to analyze development‐related endpoints such as growth, length, emergence, and reproduction with the same number of larvae (e.g., Ristola et al. 1999; Péry et al. 2002; Ducrot et al. 2004; Klagkou et al. 2024); but density‐dependent mortality is rarely studied experimentally. Hooper, Sibly, Hutchinson, and Maund (2003), for example, varied the number of larvae per test vessel with a constant total amount of food, but this resulted in a combination of interference and exploitative competition that was difficult to separate. In addition, different mechanisms, such as intraspecific mortality (e.g., by cannibalism) and starvation, may produce similar survivorship patterns under food scarcity when either only larval numbers or the food supply is manipulated in experimental studies (Fox 1975). Therefore, a two‐factorial experimental design was chosen in this study to independently investigate the effects of food supply and larval density on development, reproduction, and mortality, examining the entire life cycle from juvenile larvae to adult hatching.

As part of this study, we leveraged a mechanistic modeling approach to analyze and validate the underlying assumptions for the interpretation of the laboratory experiments with regard to the influence of food resources and larval densities. For this, we extended a previously developed dynamic energy budget (DEB) model for *C. riparius* (Koch et al. 2024), which successfully captured the physiology of individual organisms, to an individual‐based population model (DEB‐IBM) to simulate the consequences of density dependencies investigated in the laboratory experiments at the population level.

This study hypothesizes that, in addition to the already known food dependencies of development and reproduction, there is a quantitatively relevant density‐dependent interference competition with regard to mortality and emergence success in *C. riparius* . An individual‐based model will be used to quantitatively verify the experimental data and their interpretation and to test hypotheses about possible interactions between food availability and larval density. It is also intended for future use as a new module to integrate density‐dependent regulation into mechanistic population modeling of *C. riparius* .

## 2 Methods

### 2.1 Test Species

The experiments were performed with larvae from an in‐house culture of *C. riparius* , originating from a culture of Goethe University, Frankfurt am Main, Germany, in 2006. The cultures were reared in synthetic Elendt M4 medium at 20°C and a light–dark regime of 16:8 h. Freshly laid egg masses (< 1 day) were cultured in M4 medium until hatching of the larvae; these first instar larvae (< 12 h) were then added to the respective test vessels.

### 2.2 Experimental Test Design

The sediment was composed of peat powder, kaolin clay, and quartz sand seven days before the start of the experiment, according to OECD guideline 219 (OECD 2023), and the pH was adjusted by adding CaCO_3_ until a pH of approximately seven was reached. 105 g of the formulated sediment was added to each glass beaker (volume: 600 mL, diameter: 8 cm), forming a sediment layer of around 1.5 cm height. Next, 250 mL M4 medium was added to each beaker. In addition to these standard beakers of 50 cm^2^, glass aquaria with an area of 180 cm^2^ and 600 cm^2^ were filled with the corresponding amount of sediment and medium in order to obtain the same sediment‐water ratios.

After seven days of acclimation, the aeration was turned off and the desired number of larvae was randomly added to each test vessel. After 24 h, the aeration was switched on again.

The larvae were fed daily with a suspension of 1 g TetraPhyll, added to 20 mL deionized water, and homogenized with an Ultra Turrax for 2 min. Defined volumes between 10 and 1,000 μL were pipetted directly from the stirring Ultra Turrax into the respective test vessels.

**FIGURE 1:**
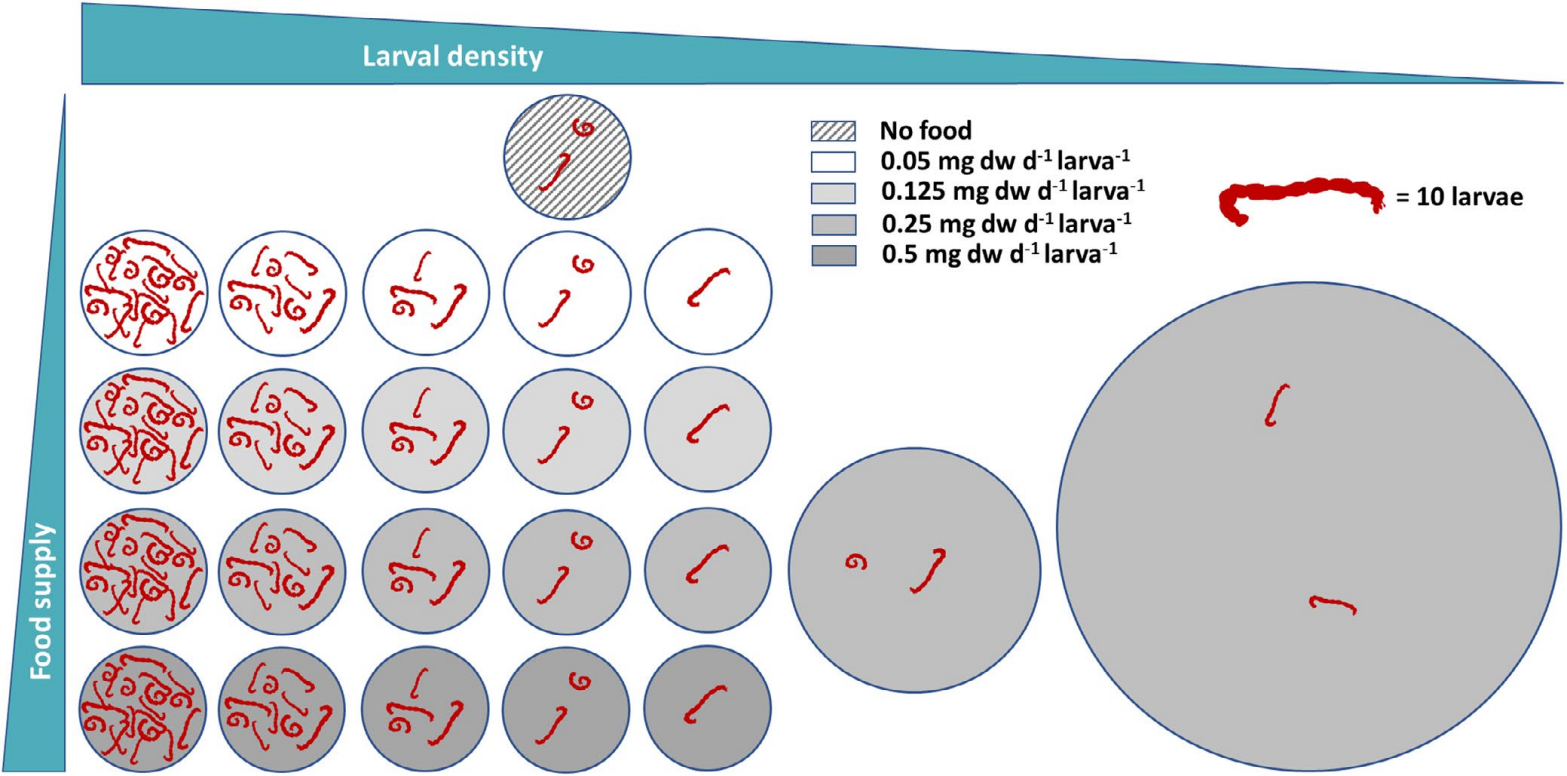
Graphical representation of the two‐factorial experimental design, which combines four food levels combined with five larval densities (10, 20, 40, 100, and 200 larvae per 50 cm^−2^ beaker), as well as two larger aquaria (180 and 600 cm^2^) with 20 larvae each at a food level of 0.25 mg dw per larva and day. A no‐food scenario was also integrated as a control. All scenarios were replicated fourfold.

The tests included a daily reference food amount per larva assuming no food limitation, set at 0.5 mg TetraPhyll larva^−1^ day^−1^ according to OECD 219 recommendations, and three lower food levels of 50%, 25%, and 10% of the reference. These food scenarios were combined with five larval densities of 10, 20, 40, 100, and 200 larvae per 50 cm^2^ beaker (see Figure 1 and Supporting Information S1, Table S1). Experiments with an increased sediment area using larger aquaria (180 and 600 cm^2^) at constant larval numbers and daily food ration per larva (20 larvae at a food level of 50% equals 0.25 mg dw d^−1^ larva^−1^) allowed the additional testing at even lower larval densities and significantly lower food availability per sediment area.

A no‐food control followed the standard design (20 larvae in 50 cm^2^ beakers) with sediment but without food addition.

Four replicates were prepared for each food‐density combination. All experiments were performed with a light intensity of 500–1000 lx and a light–dark regime of 16:8 h. No medium renewals were made during the tests, and medium losses due to evaporation were compensated for once a week with deionized water. The water temperature was recorded hourly, and oxygen content and pH values were measured once a week and at the end of the test in each test vessel.

### 2.3 Observation Endpoints

The test systems were examined daily for emerging individuals. Hatched adult midges were removed from the test vessels, and the number of emerged males and females was recorded.

To assess reproduction during the study, adult females were collected within the first day after hatching from each treatment group with the same larval density and food level, and placed into individual 400 mL glass beakers containing M4 medium of around 1 cm depth. Three male midges were added to each female for fertilization; the beakers were covered with gauze and kept under culture conditions. Egg clutches deposited in the beaker were collected individually, and the number of eggs per clutch was counted under a binocular at 100× magnification.

The replicates of the highest food levels of 0.5 and 0.25 mg larva^−1^ day^−1^ at all larval densities were each terminated after a period of 7 days without further emergence of adults, while the lower food levels were observed over the entire experimental period of 60 and 61 days, respectively. At the end of each observation period, the sediment of all replicates was examined for surviving larvae and pupae, and larvae missing compared to the initial number were classified as dead. All living larvae in the terminated replicates were dried and weighed together per replicate. The measurement results were divided by the number of larvae to calculate the mean larval dry weight per replicate.

Mortality at the end of the test was calculated as:

(1) $$\%\text{Total mortality}=\frac{\text{Initial larval number}-\mathrm{Sum}\;\text{of emerged individuals}-\text{Survivers}\;\mathrm{at}\;\text{the}\;\mathrm{end}\;\text{of the test}}{\text{Initial larval number}}\times 100$$

The daily mortality rate was calculated as:

(2) $$\text{Mean daily mortality rate}\;\left[\%d^{-1}\right]=\frac{\%\text{Total mortality}}{\text{Test duration}\;\left[d\right]}$$

Test duration is defined as the time interval between test start and the end of the respective observation period per replicate.

### 2.4 Test Validity and Physical Conditions

During all experiments, the water temperature was constant (20.3°C ± 0.3°C), and measured pH levels ranged between 6.8 and 8.4 (pH 8.0 ± 0.3).

The oxygen saturation of more than 60% required by OECD (2023) was met in all replicates up to a larval density of 100 larvae per beaker and in all replicates with 200 larvae per beaker with a daily food supply up to 0.25 mg larva^−1^. Exceptions were the highest larval density of 200 larvae per beaker combined with the highest daily food supply of 0.5 mg larva^−1^ (corresponding to 100 mg dw per beaker and day), where the oxygen saturation even dropped to values below 40% in some cases on days 14 and 19 (see Supporting Information S1).

With more than 70% emergence within 23 days at densities between 10 and 40 larvae per beaker, these experiments fulfilled the test criteria according to guideline 219 (OECD 2023) at the highest feeding level.

### 2.5 Statistical Analysis and Data Handling

Individual replicates that were significantly below the mean (M) minus the standard deviation (SD) of the four replicates per scenario and showed a deviation from the mean of more than 30% (for EmT_50_, one case) or 45% (for cumulative emergence, four cases) were treated as outliers.

For statistical analysis, the four endpoints time to 50% emergence of surviving individuals (EmT_50_) [d], daily mortality rate [% d^−1^], mortality at the end of the study [% of initial larvae], and egg number per female [# female^−1^] were each statistically tested against the amount of food per larva and larval density using multiple linear regression models (LMs) and generalized linear models (GLMs) to evaluate the data from the beaker experiments (50 cm^2^ vessels), excluding the data from the aquarium scenarios (180 and 600 cm^2^) and the starvation controls. Since no sex determination of the larvae was carried out and the model was not parameterized sex‐specifically, emerged adult males and females were also pooled for a joint evaluation of EmT_50_. All individual replicates were used for EmT_50_ statistics, while the mean of the replicates was used for statistics of the other endpoints. For both mortality endpoints, the highest feeding level (0.5 mg larva^‐1^ day^‐1^) at the highest density (200 larvae/beaker) was excluded (for rationale, see results in section 3.1.1).

All statistical analyses were performed using R 4.3.2 (R Core Team 2023). All statistical models were first tested for the significance of the interaction term for the descriptors and reduced to models without interaction terms if the interaction was not significant.

#### 2.5.1 Development

For comparability of the development in the different experimental scenarios, mortality was excluded when calculating the percentage cumulative emergence of surviving individuals over time, with:

(3) $$\%{\text{Emergence}}_{\text{surv}}=\frac{\text{Emerged adults}}{\text{Initial larval number}-\text{Dead larvae}\;\mathrm{at}\;\text{the}\;\mathrm{end}\;\text{of the test}}\times 100$$

An emergence below 100% at the end of the test period indicated surviving larvae in the test vessel.

The sigmoidal cumulative emergence survival curves over time adjusted for mortality could be well described with a two‐parameter log‐logistic model based on the Hill equation (Equation 4). This model was fitted to each cumulative emergence curve, individually, to determine the time to 50% emergence success (EmT_50_) for each replicate. The model was fitted using the function ‘drm’(settings: fct = LL.2(), type = ‘binomial’) from package ‘drc’ (Ritz et al. 2015) in R (R Core Team 2023), and was then used to interpolate the median time to emergence (EmT_50_) for each respective curve. The *R* ^2^ of all replicates' regressions was 0.979 ± 0.028 (M ± SD, *n* = 83).

(4) $$y=\frac{1}{1+{\left(\frac{{\mathrm{EmT}}_{50}}{x}\right)}^{b}}\;\left[-\right]$$

with EmT_50_: time of 50% emergence [d]; *b*: the slope of the cumulative emergence curve; *y*: fraction of emerged individuals as a function of the experimental time *x* [d].

A multiple regression model (linear model, function ‘lm’) was applied to statistically analyze the metric EmT_50_ using food level and larval density as metric descriptors. Since time to emergence data, like survival data, can not be assumed to be normally distributed and homogeneous in variances (this was also tested using Shapiro–Wilk (function shapiro.test) and Bartlett test (function bartlett.test)), we applied a non‐parametric regression approach (multiple Spearman's rank correlation).

#### 2.5.2 Mortality

Both the daily mortality rate and the mortality at the end of the study were analyzed in relation to the daily food supply per larva and the larval density.

To analyze the potential impact of excessive food on larval survival, the correlation of the daily mortality rate with the daily total food per beaker and larval density was tested.

(5) $$\text{Daily total food}\;\left[\mathrm{mg}\;{\text{beaker}}^{-1}\;{\mathrm{day}}^{-1}\right]=\text{Food}\;\mathrm{per}\;\text{larva}\;\left[\mathrm{mg}\;{\text{larva}}^{-1}\;{\mathrm{day}}^{-1}\right]\times \text{Larval number}\;\left[\text{larvae}\;{\text{beaker}}^{-1}\right]$$

Since mortality is a binomial variable, we used a generalized linear model (GLM) with beta distribution to describe the mortality rate and mortality at the end of the tests (ratio of dead to initial number of larvae) as a function of food per larva and larval density using the function ‘betareg’ from the R package betareg (Vers. 3.1.4, Cribari‐Neto and Zeileis 2010).

#### 2.5.3 Reproduction (Clutch Size)

The egg number per clutch was assumed to be Poisson distributed (discrete, [0,+inf]). Data were tested for overdispersion (function ‘dispersiontest’ from R package AER, Cameron and Trivedi 1990), and two alternative GLM models (Poisson and quasi‐Poisson, fitted with function ‚glm´ (McCullagh and Nelder 1989)).

### 2.6 Simulation Model Concept

The special focus of the present study was on density‐dependent effects resulting from competition for food and lethal stress via direct interaction, which are of fundamental importance for regulating population density. To develop and test coherent mechanistic explanations for the statistically significant empirical relationships found in this study, we used a dynamic process‐oriented modeling approach.

The individual‐based (IBM) Chironomus model used (Koch et al. 2024) was designed to simulate the population dynamics of *Chironomus riparius* in laboratory experiments, taking into account the entire life cycle from juvenile stages to emergence and reproduction. It is based on a Dynamic Energy Budget (DEB) model, describing the physiology of chironomid individuals, and was extended to also consider the interaction of individuals at the population level. Newly introduced parameters at the population level were calibrated using laboratory experiments described in this paper. This extended model considers two types of interactions: (a) direct interference competition between the individuals leading to density‐dependent mortality at higher population densities and (b) indirect exploitative competition for food. Thus, increased mortality can also have indirect favorable effects on individual development by increasing the food supply for the surviving larvae. The overall model was then tested using independent literature data. The IBM Chironomus population model (version 2.1) was implemented in Delphi Embarcadero 2010 RAD Studio XE2; the full model equations are shown in the ODD (Objects, Design concepts, and Details) protocol in the Supporting Information.

### 2.7 Modeled Spatial and Temporal Scales

With the population model, we aimed to simulate the experimental setup, thus setting up a total area of 0.5 m^2^ as the habitat size of the modeled population. The total area was arranged as a rectangular grid map and consisted of 20 individual cells with an area of 0.025 m^2^, within which the larvae can migrate. Each cell differs in terms of the number of larvae and the amount of food. The density of the simulated population results from the sum of the individuals divided by the total sediment area.

Here, we used discrete time steps in the implementation of the DEB model: The food uptake of each larva was calculated hourly and summed up over 24 h of the day. All other processes, such as growth and development, emergence of the adults, mortality, and movement, were calculated in daily steps.

### 2.8 Individual Level Submodules

#### 2.8.1 The Physiological DEB Model

Individual organisms were modeled using the Dynamic Energy Budget (DEB) theory (Kooijman 2009). DEB models describe all individual life‐history processes based on energy fluxes: Organisms assimilate resources from their environment and subsequently allocate energy through a reserve compartment to maintenance, somatic growth, and the reproduction system. In the reproduction system, maturity is built up over time until a predetermined threshold for maturity is reached (termed puperty). After that, maturation ends, and investment in offspring begins. Metabolic processes over time depend fundamentally on the energy availability, the temperature, and the size of the individual.

Here, we used the DEB model variant *hax* for holometabolous insects (Llandres et al. 2015; Kooijman 2010) to simulate the whole life history of *C. riparius*. The *hax* model is characterized by a phase of metabolic acceleration (and accelerated growth) between birth and the start of investment in reproduction, as well as the additional life stage transitions of pupation and emergence. A complete model description of the *hax* model and the physiological DEB parameters optimized to reproduce life history data of *C. riparius* at different temperatures can be found in Koch et al. (2024).

#### 2.8.2 Survival

To simulate larval mortality in the laboratory experiments, only intraspecific density‐dependent mortality and starvation‐induced mortality through complete depletion of an individual's reserve compartment were taken into account. Since any background mortality that may have occurred in the experiments was already included in the density‐dependent mortality, no separate background mortality was assumed.

#### 2.8.3 Movement

Since a higher preference for high‐quality food is the main driver of habitat selection of *C. riparius* (de Haas et al. 2005), a simple food‐dependent movement module was implemented. The movement of each individual from its current cell to a randomly selected neighboring cell within the grid map (according to von Neumann's 4‐neighborhood rules) is determined by the individual's energy state: Once a day, an individual moves to a neighboring cell if a randomly generated number exceeds its calculated probability of staying, which depends on the individual's energy level (scaled reserve density, e [−]). Hence, the probability of larval movement increases in the model when energy reserves are low.

### 2.9 Population‐Level Submodules

#### 2.9.1 Density‐Dependent Mortality

**FIGURE A1:**
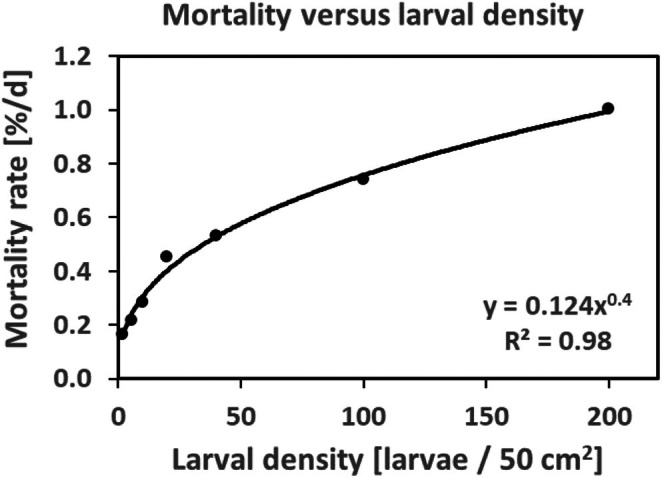
Experimentally derived equation for the density‐dependent mortality rate [% day^−1^] at a constant food level of 0.25 mg larva^−1^ day^−1^.

The model assumes a fixed, density‐dependent mortality rate that acts continuously over time and depends only on larval density, and was derived from the data generated in this study. Based on an experimentally derived regression between the initial larval density and the mean mortality rate at a feeding level of 0.25 mg larva^−1^ day^−1^ (see Figure A1 in the Appendix A), the daily mortality rate can be described by the power function *y* = *a* x^0.4^ [% d^−1^] as a function of the larval density × [larvae per area]. The mortality coefficient *a* was determined by calibration (see Section 2.11). The susceptibility of each larva to be killed as a result of density‐dependent mortality is determined by a uniformly distributed individual random number between 0 and 1, which is renewed daily.

#### 2.9.2 Food Availability and Uptake

In the experimental study, the daily food addition at the beginning of each day [mg dw m^−2^] was given as the product of the initial larval density per scenario [larvae m^−2^] and the individual food supply [mg dw larva^−1^ day^−1^].

The potentially available food energy on the sediment surface X [J m^−2^] for ingestion by the chironomids depends on the actual energy content of the fresh food, the energy loss due to microbial degradation, and the reduced availability of food due to mixing into the sediment. The latter aspect, in particular, can lead to a strong reduction in the energy available to the larvae, as *Chironomus riparius* only consumes food available directly upon the sediment surface (Naylor and Rodrigues 1995). However, it should also be noted that the calorimetric energy requirement of *C. riparius* in the used DEB model was previously calibrated indirectly based on growth data and not with actual food ingestion data (Koch et al. 2024). The modeled ingestion rates can therefore deviate slightly from the real ingestion rates. To compensate for all these environmental and model uncertainties, a food conversion factor F_C_ [J mg dw^−1^] was calibrated as a free model parameter. It is expressed in Joules, but is subject to the same error (positive or negative) as the ingestion rate parameter of the DEB model, thus ensuring internal consistency within the modeled system.

This results in:

(6) $$\text{Daily energy addition}\;\left[J\;m^{-2}\right]=\text{Larval density}\;\left[\text{larvae}\;m^{-2}\right]\times \text{Individual food supply}\;\left[\mathrm{mg}\;{\text{larva}}^{-1}\;{\mathrm{day}}^{-1}\right]\times F_{C}\;\left[J\;\mathrm{mg}\;{\mathrm{dw}}^{-1}\right]$$

When reproducing the experimental setup in the model, the supplied food (expressed in J) was allowed to accumulate on the sediment surface over time without further degradation and was only reduced by the food consumption of the larvae.

The larvae were feeding every hour until the end of the day or until the food was depleted. If the food was no longer sufficient to cover the intake of all larvae within one time step, the available food was distributed among the larvae in proportion to their potential intake rate.

The actual feeding (food uptake) per individual depends not only on temperature and larval size, but also on food availability per sediment area, which is described by a Holling type II functional response *f(X) =* $\frac{X}{K_{S}+X}$ (with: *K*_*s*_: half‐saturation constant for food uptake [J/m^2^]; *X*: food density [J m^−2^]).

While all other species‐specific parameters were derived from the existing DEB model (Koch et al. 2024), the half‐saturation constant *K*_*S*_ for the functional response *f(X)*, i.e., the resource availability at which half of the maximum uptake is reached, was calibrated in this study.

### 2.10 Model Stochasticity

To reproduce the sigmoidal cumulative emergence pattern in the population‐level experiments, inter‐individual variability of development time was integrated into the model. To estimate the individual variability, we followed the approach of Koch and De Schamphelaere (2020), who added the variability to the maximum assimilation rate ${\dot{p}}_{\mathrm{Am}}$. Based on laboratory control data for *C. riparius* emergence without food limitation, a log‐normal distribution with a standard deviation 𝜎 of 0.35 delivered the best overall fit (for details, see supporting information in Koch et al. 2024). At birth, a fixed individual value for ${\dot{p}}_{\mathrm{Am}}$ is calculated and assigned for each individual.

An additional, albeit less influential source of variability lies in the random initial distribution of the individuals across the spatial grid cells and their subsequent movement between the cells.

All simulation runs were carried out with 20 Monte Carlo simulations to compensate for the variability of each run (i.e., to obtain a robust mean value). The resulting model variability of the total emergence in Monte Carlo simulations depending on larval density and food quantity is shown in the Supporting Information S1.

### 2.11 Model Parameterisation and Calibration

To the existing DEB model for chironomids (Koch et al. 2024), we added new extensions only for food dependency and density‐dependent mortality. This resulted in three new parameters to be calibrated: the half‐saturation constant K_S_ for food uptake, the density‐dependent mortality rate coefficient a, and the food conversion factor F_C_. All three parameters were calibrated simultaneously using the measured cumulative emergence (sum of males and females) over time as calibration data by applying the deterministic method of downhill simplex optimization (Nelder and Mead 1964; Press et al. 1989).

All data sets were used for parameter calibration, except the scenarios at 200 larvae per beaker, to save computing time due to the very high larval numbers in these scenarios. During calibration, the scenario with the lowest larval density in 600 cm^2^ aquaria was weighted 8 times higher than the 16 scenarios with 50 cm^2^ beakers at densities of up to 100 larvae per beaker to ensure that this data set, which is essential for the calibration of the half‐saturation constant K_S_, had a sufficiently large influence on the calibration results and that the model results reflect larval development in this aquarium scenario as realistically as possible.

### 2.12 Initial Model Conditions

During initialization, the DEB state parameters were set according to the parameter values for ‘maturity at birth’ (Koch et al. 2024). This involves calculating the state parameters of each individual based on DEB rules. Throughout this process, we assume that the individuals have no energy constraints. The initialization routine is completed as soon as the individuals have reached their initial size of newly hatched larvae.

Finally, an individual value for the individual correction factor of the maximum assimilation rate ${\dot{p}}_{\mathrm{Am}}$ [J/d] is sampled for each new larva from a log‐normal distribution as described above.

The simulations are initiated by randomly distributing the scenario‐specific initial larval density [larvae m^−2^] to all cells within the grid map. The initial food energy density of all cells was set to 0 J m^−2^.

### 2.13 Model Validation

We used laboratory data from Hooper, Sibly, Hutchinson, and Maund (2003) to validate our model and specifically the hypothesis of density‐dependent mortality as observed in our experiments. In their study, two experiments were carried out with *C. riparius* larvae (1–2 days after hatching) to investigate survival, fecundity, and growth at different larval densities under constant laboratory conditions. Both experiments were conducted at 20°C using Aquarian Tropical Fish Flakes as a food source. Experiment 1 started with 50 larvae in aquaria of 600 cm^2^ with food additions between 25 (low food scenario) and 75 (high food scenario) mg aquarium^−1^ d^−1^. Emergent adults were able to mate and reproduce within the covered aquaria over an observation period of 35 weeks.

In experiment 2, larval densities of 1, 1.7, 4, 8, 16, and 35 larvae cm^−2^ were incubated in beakers with a size of 127.5 cm^2^ at a constant food addition of 50 mg beaker^−1^ d^−1^ for 56 days; the emerging success of the larvae within this experimental phase was recorded.

To simulate these experiments, only the respective larval densities, feeding conditions, and container sizes were adjusted. The little information available on the composition of TetraPhyll Flakes (manufacturer's information on protein and fat content: 46% and 11%, calorimetric value: 19.9 kJ/g (Müller et al. 2013)) and Aquarian Tropical Fish Flakes (manufacturer's information on protein and fat content: 35% and 11.9%; metabolisable energy 16.7 kJ/g (Priestley et al. 2006)) shows that the energy content of the respective fish food is of a comparable order of magnitude in both studies.

## 3 Results

### 3.1 Experimental Results

#### 3.1.1 Development and Cumulative Emergence

None of the individuals in the no‐food controls emerged, and mortality was 83.7% ± 14.3% at the test end. Very little growth of the surviving larvae was observed in the no‐food controls over 60 days (see Supporting Information S1).

**FIGURE 2:**
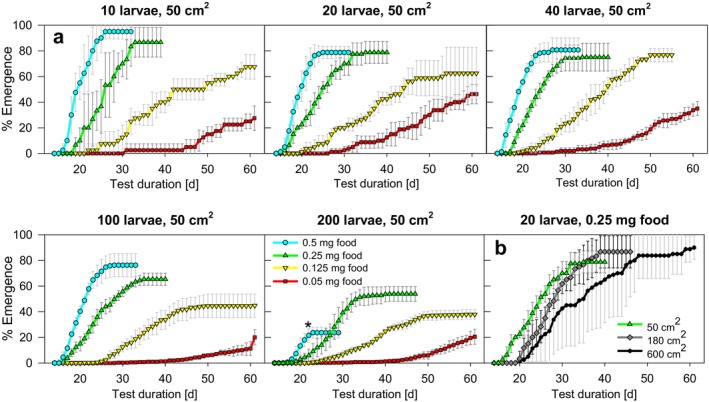
Cumulative emergence success in the beaker experiments (a) as a function of larval density (10, 20, 40, 100, and 200 larvae per 50 cm^2^) and four food levels (0.05, 0.125, 0.25, and 0.5 mg food larva^−1^ d^−1^), and (b) as a function of sediment area with 50, 180 and 600 cm^2^ (20 initial larvae per aquarium 0.25 mg food larva^−1^ d^−1^). M ± SD of replicates. *Data not used for further analysis.

Broadly, in treatments with regular feeding, the cumulative emergence over time (pooled data of hatched adult males and females) showed a clear dependence of the development rate on the amount of food in all experiments (Figure 2a). Lower emergence rates at the end of the experiment with higher larval density and reduced food can also be seen (Figure 2; for sex‐specific emergence see Supporting Information S1).

After an initially high emergence rate in the scenario with the highest total food supply and larval density (0.5 mg food larva^−1^ day^−1^ and 200 larvae per beaker), there was a clear decline in hatching success from day 20 onwards. This is attributed to the consequences of the very high food supply per beaker, which likely led to low oxygen levels and high larval mortality. As severe damage to the larvae at high food density due to oxygen deficiency and waste accumulation (Beaty 1995) cannot be ruled out in our experiment. According to these findings, this data set was no longer included in the evaluation of density‐dependent mortality.

Development time was longer also with increasing size of the test systems (180 and 600 cm^2^) compared to the beaker experiments with the same number of larvae and addition of food, which resulted in lower food densities per area (Figure 2b).

**FIGURE 3:**
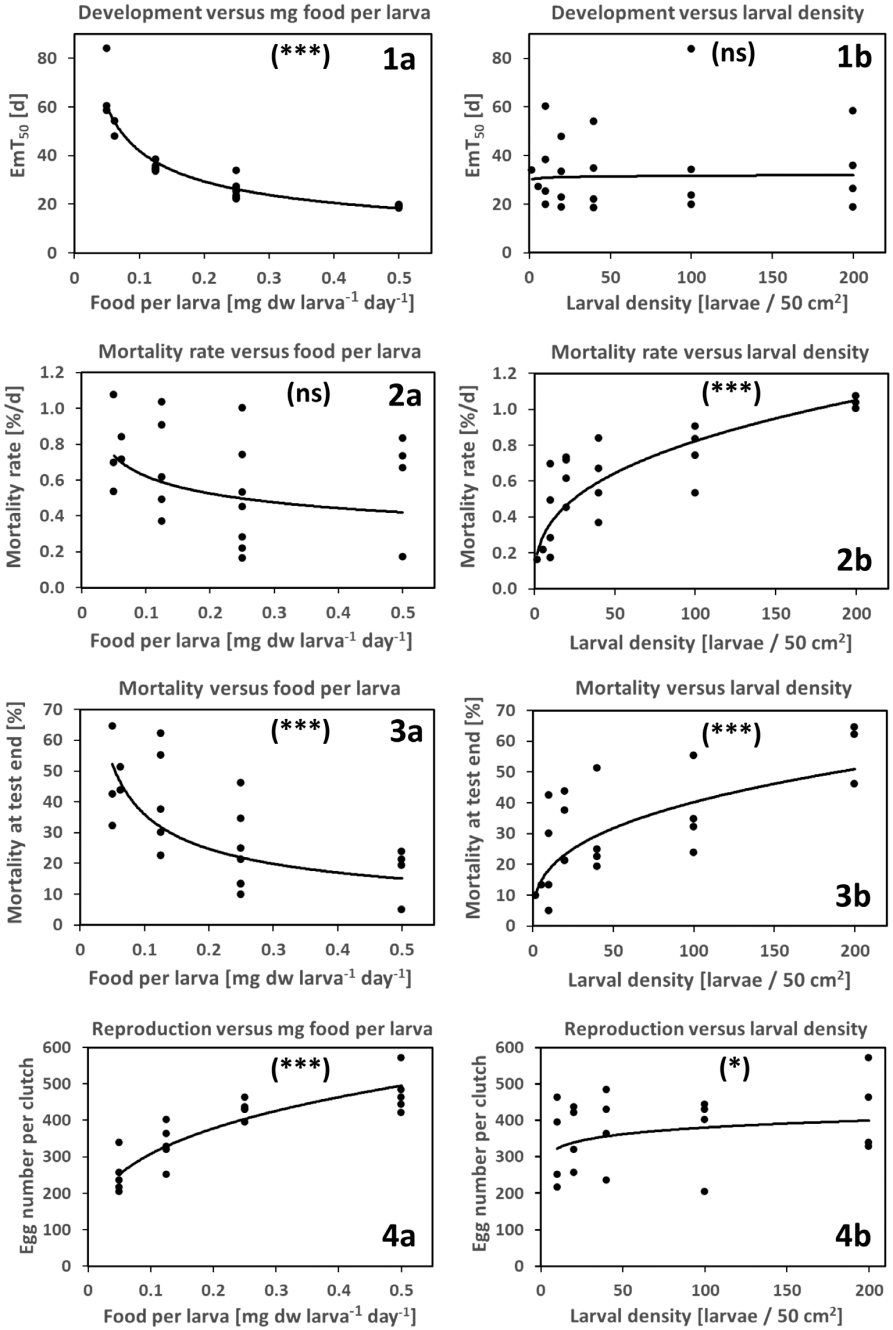
Regression between (1) EmT_50_, (2) mortality rate [%/d], (3) mortality at test end [% of the initial number of larvae], (4) egg number per clutch, and food per larva (left, a) or larval density (right, b). Significance level of LM and GLM statistics in brackets. ns, not significant, *p < 0.05; **p < 0.01; ***p < 0.001. Trend model: *y* = ax^b^ (trend model coefficients are given in the Supporting Information S1).

The time to emergence of 50% of the surviving individuals (EmT_50_) [d] showed a significant negative correlation with food per larva (*p* = 2.22e‐14, see Figure 3, 1a), but no significant correlation with larval density (*p* = 0.42, see Figure 3, 1b). No significant interaction was found between food per larva × larval density (*p* = 0.451).

#### 3.1.2 Mortality

Two mortality endpoints, the daily mortality rate (independent of the test duration) and the mortality at the end of the respective test duration, were evaluated independently. The daily mortality rate [% d^−1^] correlated significantly with larval density (*p* = 2.07e‐05, see Figure 3, 2b), but not with the daily food per larva (*p* = 0.355, see Figure 3, 2a). No significant interaction was found between food per larva × larval density (*p* = 0.125). There was also no significant correlation between the daily mortality rate and the total daily food supply per beaker (*p* = 0.704), while the correlation with the larval density was again positively significant (*p* = 0.00095). No significant interaction was found between total food per beaker × larval density (*p* = 0.452). Hence, negative effects on larval survival due to both an increase in the total amount of food per beaker (e.g., as a result of unfavorable conditions caused by excessive food enrichment) as well as a decrease in the total amount of food can therefore be excluded. The one combination with the highest number of larvae and the highest food per capita, which may have affected mortality in the experiment, was previously removed from the data set used in this statistical analysis. Mortality at the end of the tests proved to be positively correlated to larval density (*p* = 3.43e‐06, see Figure 3, 3b), but also negatively correlated to food per larva (*p* = 5.84e‐07, see Figure 3, 3a). No significant interaction was found between food per larva × larval density (*p* = 0.333).

#### 3.1.3 Reproduction

**FIGURE 4:**
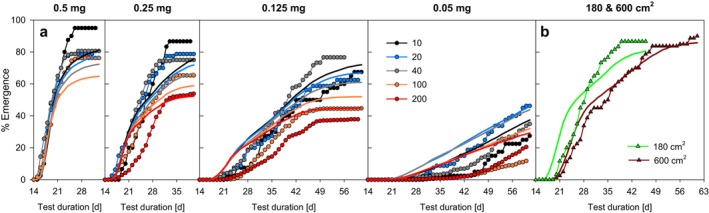
Cumulative emergence success in measured data (mean values of replicates, symbols) and simulations (mean of 20 Monte Carlo simulations, simple lines). (a) Different larval densities (10, 20, 40, 100, and 200 larvae per 50 cm^2^) per food level [mg food larva^−1^ d^−1^]. (b) Enlarged sediment area with 180 and 600 cm^2^ (20 initial larvae per aquarium at 0.25 mg food larva^−1^ d^−1^).

As the egg numbers proved to be highly overdispersed (*p* = 5.641e‐05), the quasi‐Poisson distribution was used for further analysis. Egg number per clutch proved to be significantly positively correlated to food per larva (*p* = 1.07e‐05, see Figure 3, 4a), and to a much lesser extent to larval density (*p* = 0.0477, see Figure 3, 4b). No significant interaction was found between food per larva × larval density (*p* = 0.832).

### 3.2 Simulation Results

To simulate the density dependence experiments, three new model parameters had to be calibrated simultaneously in addition to the parameters already given in Koch et al. (2024), using the measured cumulative emergence of the entire data set except for the 200‐larvae treatments, and were reported as mean values ± SD of 12 independent calibration runs: (i) the calibrated food conversion factor F_C_ for the amount of food supplied experimentally to the energy available to the larvae in the model is 2.3 ± 0.14 kJ g dw^−1^ TeraPhyll, assuming a constant assimilation efficiency $\kappa_{X}$ of 0.8 of the ingested food in the DEB model, which applies to all food supply scenarios tested, (ii) the half‐saturation constant for feeding *K*_*S*_ of 1045 ± 217 J m^−2^, and (iii) the mortality rate coefficient (a) for the power function *y* = a x^0.4^ [% d^−1^]: a = 0.29 ± 0.02 for larval density per 50 cm^2^, corresponding to a value of 0.035 for larval density per m^2^.

In general, the simulations showed a good overall match with the sigmoid cumulative emergence patterns found in the experiments over time, which validates the assumed log‐normally distributed individual variability of larval feeding and thus their individual growth in the model. This applies to the standard experiments with 50 cm^2^ beakers (Figure 4a) as well as to the large aquaria up to 600 cm^2^ (Figure 4b), in which the larval development rate is additionally slowed down by the reduced food density per sediment area, which could be adequately simulated by the calibrated functional response of food intake. The cumulative emergence data corrected for mortality in experimental data and simulation results also match well (see Supporting Information S1, Figure S7). However, the simulated cumulative emergence in the later phase of the experiments is slightly too low when the food supply is high, while it begins slightly too early at lower food levels (Figure 4a).

Compared to simulations without any food limitation (see Supporting Information S1, Figure S8), the development time EmT_50_ [d] is extended by 1.05 ± 0.17 days in the scenarios of 0.5 mg larva^−1^ d^−1^. This indicates an already obvious, albeit still minor, food limitation in the simulations of the highest food supply.

**FIGURE 5:**
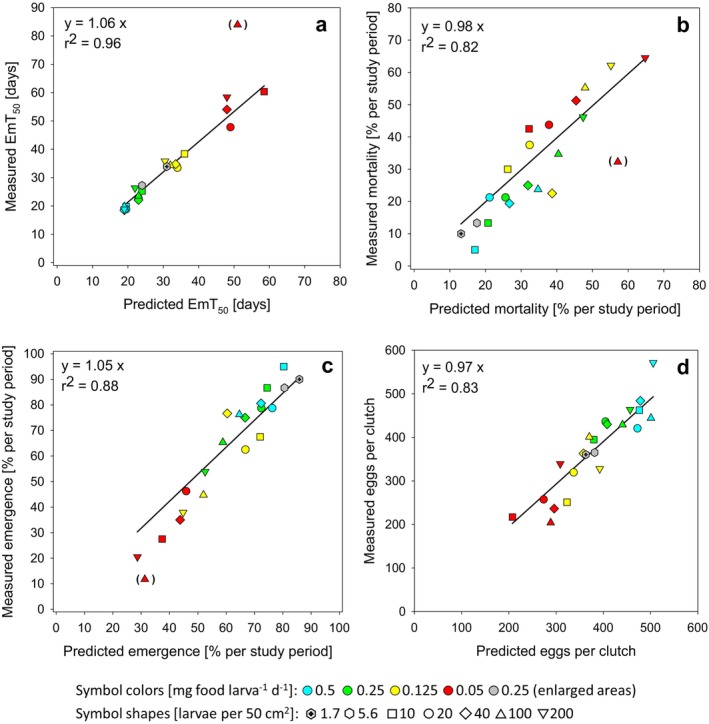
Correlation of model‐based predicted versus measured values: (a) EmT_50_ (data corrected for mortality), (b) % mortality at test end, (c) % emerged adults at test end, (d) mean eggs per clutch (red triangles in brackets are considered as experimental outliers and were not included in the regressions).

The direct comparison of the simulation results shows a good agreement with the experimental observations for all four measured endpoints (Figure 5).

As a function of food supply per larva, clear clusters with similar development rates can be found independent of larval density (EmT_50_, see Figure 5a), with some obvious exceptions. In the scenarios with the lowest total food supply per area (≤ 0.1 g dw m^−2^ d^−2^, see Table S1), that is also considerably below 50% of the half‐saturation constant K_S_ for feeding, an extension of the development time is observed, which is correctly reproduced by the model. This is most evident in scenarios with very low larval densities (e.g., food quantity of 0.25 mg larva^−1^ day^−1^ in large aquaria (600 cm^2^) with 20 initial larvae) or very low food levels per larva (food quantity of 0.05 mg larva^−1^ day^−1^ in small beakers (50 cm^2^) with 10 larvae, see Figure 5a). In the lowest food scenario (0.05 mg food larva^−1^ day^−1^) at densities of 20, 40, and 200 larvae per beaker, there is obvious scatter between the experimentally derived EmT_50_ values at similar simulation values, with a slight increase in experimental EmT_50_ values at higher larval densities. It is unclear whether these deviations are due to experimental variability or model deficits under these specific feeding conditions. Model inaccuracy under low food conditions is rather unlikely, as simulations were able to reproduce measured data well at even lower food availability at 10 larvae per beaker and in the aquarium experiments (see above).

Both the increase in mortality (Figure 5b) with increasing larval density within each food level, and the increase in mortality with decreasing food quantity is well reproduced by the model. In accordance with the data, the simulations of the aquaria (180 and 600 cm^2^) with the lowest larval density are in the lowest mortality range. In the model output for these scenarios, death by starvation would only occur below a food supply of approximately 0.005 mg larva^−1^ d^−1^ (determination of this threshold value was carried out with density‐dependent mortality switched off). Under the feeding conditions of our laboratory experiments, no starvation‐related mortality occurred in the model results, and density‐dependent mortality was the only cause of mortality.

The number of emerging individuals within the respective test period (Figure 5c) is additionally reduced, particularly at the low food level of 0.05 mg larva^−1^ day^−1^ due to a high proportion of larvae still alive at the end of the experiment.

For the analysis of reproduction, between 29 (at 0.05 mg larva^−1^ day^−1^) and 117 egg ropes (at 0.5 mg larva^−1^ day^−1^) could be evaluated per food level, as well as 17 and 18 ropes in the two aquarium experiments, respectively. The DEB model was able to reproduce the food dependence of clutch size quite well (Figure 5d, see also Supporting Information S1, Figure S9), including the lower fecundity in the aquarium scenarios compared to the higher egg numbers at the same food level of 0.25 larva^−1^ day^−1^ studied in smaller beakers.

**FIGURE 6:**
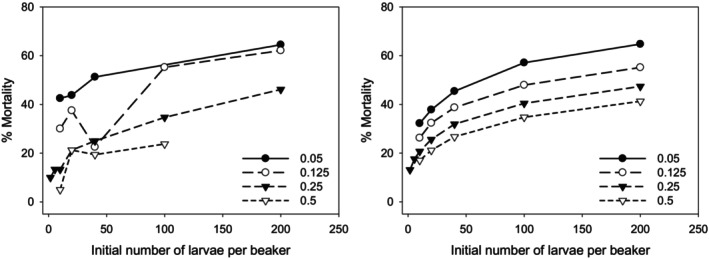
Measured (left) and simulated (right) dependence of mortality [%] at the end of the experiments [%] on the initial larval density per beaker (50 cm^2^) and food level ([mg larva^−1^ day^−1^], different lines).

The dependence of mortality on larval density and food levels examined is summarized in Figure 6. In addition to the positive correlation of larval density with the mortality rate, another aspect is evident both in the experiments and in the simulations: the higher the food quantity, the lower the effective mortality at the end of the test.

#### 3.2.1 Model Validation

**FIGURE 7:**
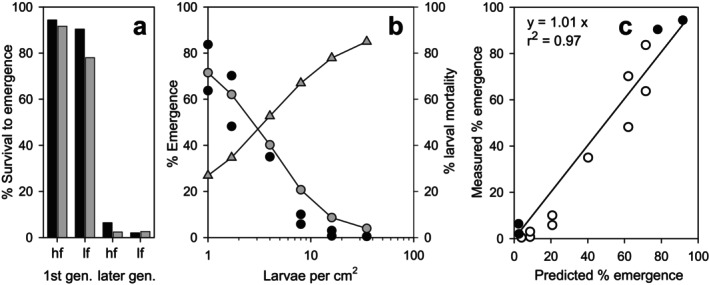
Comparison of experimental data (Hooper, Sibly, Hutchinson, and Maund 2003) and simulations. (a) Experiment 1 (long‐term study in aquaria). Survival to emergence [%] (black: measured; gray: simulated), separated for the first generation and the following generations from week 5 to 34 at two food levels (hf:high food; lf: low food). (b) Experiment 2 (56‐day study in beakers). Measured (black dots) and simulated (gray dots) percentage of cumulative emerged larvae within 56 days. Gray triangles: simulated % density‐dependent larval mortality. (c) Correlation between predicted and measured percentage of emerged larvae in experiment 1 (black dots) and experiment 2 (white dots).

Data generated in two experiments conducted by Hooper, Sibly, Hutchinson, and Maund (2003) were used to verify our modeling concept. While our experiments are designed to maximize the independence of the variables larval density and food supply per larva, an increased larval density in the experiments of Hooper, Sibly, Hutchinson, and Maund (2003) leads to a significantly lower food supply per larva due to the constant food supply per vessel. Overall, our model could reproduce these independent data very well (*R* ^2^ = 0.97 for a linear regression between predicted and measured emergence data, see Figure 7). In experiment 1, the first generation had a starting density of 0.08 larvae cm^−2^, distributed over the 600 cm^2^ sediment surface of each aquarium, and a food supply of 0.5 and 1.5 mg larva^−1^ d^−1^. During this experiment, the simulated larval densities after week 17 occasionally reached up to 6 (low food) and 10 (high food) larvae cm^−2^, resulting in extremely low food availability per larva (< 0.01 mg larva^−1^ d^−1^). The simulations reflect well the productivity in the high and low food supply scenarios in comparison with the experimental data in weeks 17 to 35 for emergence (80%–125% of the measurements) and the sum of eggs per aquarium (90%–75% of the measurements). The order of magnitude of the measured and simulated proportion of successful emergence under very different conditions in terms of food availability and larval density for the first generation and subsequent generations is quite comparable (Figure 7a). In particular, the high mortality rate of over 95% of a self‐limiting population with very high larval densities could be reproduced well.

In experiment 2, the uniform daily food addition to the test beakers at initial densities of between 1 and 35 larvae cm^−2^ resulted in a per capita food supply of 0.391 down to 0.011 mg fish food larva^−1^ d^−1^. The observed percentage emergence rate of the larvae over the test period of 8 weeks was consistent with the model predictions up to a density of 4 larvae cm^−2^, and the corresponding mortality was within the range of our own experiments (Figure 7b). At higher densities, only a slight overestimation of the simulated emergence rate was visible, and a mortality of up to 85% was achieved. Simulating these scenarios, starvation did not occur in the model, and density‐dependent mortality remained the only cause of mortality.

Figure 7c shows a comparative overview of both experiments, in which hatching successes of less than 3% up to over 90% could be well reproduced with the model under the different test‐specific density and feeding conditions as well as test durations.

## 4 Discussion

This study aimed to identify and quantify mechanisms regulating populations of *Chironomus riparius* . In systematic experiments with a separation of food supply and larval density, it can be shown that chironomid populations are regulated via two different routes. The first one is via exploitative competition and food‐limited development and egg production. The other one seems to be a more direct mechanism where larval mortality is positively correlated with larval density, indicating intraspecific lethal interactions. In addition, an existing DEB‐based model was extended to include these processes, calibrated using these experimental data, to test the plausibility of the mortality assumptions made in this study, and then validated with independent data from the literature.

### 4.1 Impact and Interaction of Food Supply and Larval Density

In our experiments, larval development, i.e., time to emergence, was clearly related to food supply. However, no indication was found that development time is negatively affected by larval density by itself, i.e., there is no evidence for a density‐dependent inhibition of developmental rate in these experiments up to the maximum tested density of 4 larvae cm^2^. The reproduction rate as the number of eggs per female is also clearly related to food, with mean egg numbers at the highest feeding level of 476 eggs per female, which is in line with experimental results from Péry et al. (2002), Ducrot et al. (2004), and Klagkou et al. (2024), who found similarly high egg numbers at ≥ 0.6 mg TetraMin larva^−1^ d^−1^ (410, 425, and 420 eggs per female, respectively). We also found a slight positive correlation between egg numbers and larvae density. This is consistent with the results of Ristola et al. (1999), who found a slight increase in growth rate in *C. riparius* with increasing larval density, possibly caused by excess food per larva at high larval numbers or increased food density on the sediment due to the increased total amount of food. In our experiments, the increasing mortality at higher larval densities during the experimental period may have led to a higher food supply per surviving larva, as daily feeding per beaker was kept constant. However, this density‐dependent promoting effect caused by the experimental design is relatively small and could only be demonstrated for clutch size.

Larval mortality rates of up to 1% per day determined in this study were driven only by larval density, not food supply, suggesting that density‐dependent mortality acted directly via intraspecific interference competition. As the mortality rate occurred daily, the resulting number of dead larvae also depended on the development time. This was clear from the negative correlation between food supply and effective total mortality at the end of the study: Food shortage can slow down the development of larvae and prolong the period of vulnerability to intraspecific mortality. Thus, food supply has an indirect effect on the effective mortality, and the highest mortality occurs under high larval density and low food conditions.

For *Chironomus tentans* , Lo (1996) found in laboratory experiments that a longer exposure time to conspecifics increases the possibility of encounters and, therefore, the risk of being cannibalized. A higher intraspecific mortality at lower growth rates has also been reported for the cannibalistic dipteran predator *Chaoborus crystallinus* (Strauss et al. 2016) and for several fish species (Accolla et al. 2023). This combined effect of food and density on mortality appears to be an important mechanism for characterizing intraspecific losses in populations not only under laboratory conditions but also in the field.

### 4.2 Mechanisms Behind the Density‐Dependent Larval Mortality

The food‐independent but density‐dependent loss of larvae observed in our experiments is regarded as intraspecific mortality by interference competition. *C. riparius* is not an obligate predator; predation has been documented only exceptionally (e.g., Pillot 2014). However, cannibalism in insects is not restricted to carnivorous species and also occurs in herbivores and in detritivores (Richardson et al. 2010). In the literature, cannibalism in *C. riparius* is occasionally mentioned in laboratory tests without specifying the mechanism more precisely (e.g., Azevedo‐Pereira et al. 2012; Carrasco‐Navarro et al. 2021). Lo (1996) describes a density‐dependent mortality in *Chironomus tentans* and considers missing larvae at the end of the experiments to be victims of cannibalism. In contrast, Beaty (1995) attributes the absence of larvae in *C. riparius* to the consumption of dead animals by conspecifics and leaves the question of active cannibalism open. Own observations with 25 different‐sized *C. riparius* larvae in Petri dishes (6 cm diameter) over 96 h without sediment and food revealed that about 6% of larvae were eaten per day at an initial density of approximately 1 larva per cm^2^ (T. Strauss, pers. comm.). It could be observed that the larvae actively met and attacked each other with their mouthparts, and nibbling on conspecifics apparently led to lethal injuries. The fact that larvae were ultimately killed by these attacks could not be observed directly, but is very likely. Dead larvae were eaten from back to front by several larvae within a few hours, leaving only the victim's head at the end. So far, it is impossible to distinguish between deliberate cannibalism and necrophagy after prior killing of the victims, and there is no reliable evidence of ingestion or metabolization of the victim's tissue. However, these observations support the assumption that the mechanism of density‐dependent mortality in *C. riparius* is the aggressive killing of conspecifics. Egg cannibalism does not play a role in our experiments, as we used already hatched larvae. Polis (1981) also emphasizes that cannibalism may also occur as a by‐product of the normal feeding activities of some species, including deposit feeders, and that crowding increases the frequency with which conspecifics violate a critical minimum distance, thus promoting an increase in cannibalism rates at high densities.

### 4.3 Model Performance at Low Food Supply and High Larval Density

Overall, the individual‐based DEB model for *C. riparius* was able to reproduce the experimental results very well. On the one hand, the DEB model of Koch et al. (2024) is well applicable to the physiological, food‐driven conditions in the experiment and enables a good model‐based prediction of the measured food‐dependent development delay solely through a proportional reduction in food supply per larva without further changes to life history parameters in the model. On the other hand, the integration of the density‐dependent mortality calibrated to the experimental conditions allows us to confirm our hypotheses on effective mortality as a function of the density‐dependent mortality rate interacting with the food‐dependent development rate.

Despite the high importance of half‐saturation constants in functional responses for the understanding of ecosystem functions, robust empirical data are limited to a few taxonomic groups (Mulder and Hendriks 2014). The inclusion of the low food addition aquarium experiments in our study made it possible to calibrate the half‐saturation constant K_S_ for food uptake for the functional response for *C. riparius* , which thus allows the model to realistically simulate growth inhibition even at very low area‐related food densities. Therefore, the *C. riparius* population model is applicable at high larval densities via the density‐regulated mortality as well as at low food availability via the functional response. The experiments reported by Hooper, Sibly, Hutchinson, and Maund (2003) served as an additional validation of our modeling approach and, due to the good agreement of the model results with these experiments, confirms our assumption that the mortality rate depends on the larval density, and that the effective mortality is influenced by food‐dependent development time and thus by the duration of potential vulnerability.

### 4.4 Inter‐Individual Variability

In order to realistically simulate temporal scattering within emergence peaks as well as temporal overlaps of emergent cohorts in the field, the implementation of inter‐individual variability in the model is an essential prerequisite. In this laboratory study, the individual variability derived from sigmoidal cumulative emergence curves over time in food‐saturated experiments (Koch et al. 2024) could be successfully transferred to different food‐level scenarios without further model adjustment.

### 4.5 Comparison of Experimental and Field Larval Densities

Maximum field densities from 1.7 to 4 larvae cm^−2^ (Gower and Buckland 1978; Koehn and Frank 1980; Davies and Hawkes 1981) have been reported for *C. riparius* under favorable conditions, but higher density ranges from 5 to over 10 larvae cm^−2^ are also quite possible (Edwards 1957; Rasmussen 1984; Groenendijk et al. 1998). Such high abundances often occur in organically polluted conditions (Gower and Buckland 1978; Pillot 2014), with sewage effluent discharge promoting *C. riparius* (Davies and Hawkes 1981). The upper range of the tested densities in our experiments of 0.034–4 larvae cm^−2^ is in the middle range of these maximum field densities. The densities in the two experiments by Hooper, Sibly, Hutchinson, and Maund (2003), which we used for model validation, range from 0.08 to 35 larvae cm^−2^. Thus, the analyzed and modeled densities cover the maximum expected abundances for *C. riparius* in the field.

According to our experimental results and the simulations confirming them, the high densities observed in the field must already be subject to massive density regulation by interference competition, which prevents further overshooting into even higher population densities. Due to their often very high biomass productivity (Benke 1998) and high relevance as a food source for many predators (Armitage 1995), emerging chironomids make a major contribution to trophic energy transfer for higher trophic levels, especially in terrestrial food webs (Kautza and Sullivan 2016). Therefore, knowledge of density‐dependent loss rates as a regulating mechanism of population dynamics is essential for understanding trophic dynamics, especially at high abundances at the capacity limit.

### 4.6 Importance of Long‐Term Experiments

In our experiments, we were able to detect mortality rates of over 60% at high larval densities over a period of two months, which should have a significant impact on the population dynamics of *C. riparius* . In contrast, in studies using standard test protocols (e.g., OECD 2023, laboratory test with 20 larvae per replicate in 50 cm^2^ beakers), a density‐specific daily mortality rate of about 0.85%–1.0% would theoretically cause a mortality of less than one larva in a 4‐day test (< 4%), and below four larva within a test period of 21 days (< 20%); and is therefore neither conspicuous nor statistically robustly quantifiable. However, low mortality is one crucial study validity criterion. Therefore, when realistic and more robust estimates of mortality rates are of interest, especially when density dependence is evaluated, we recommend longer‐term experiments.

### 4.7 Recommendation for Non‐Tested Species

However, if no empirical data are available for other species of interest, which would usually only be generated with considerable experimental effort, density‐dependent regulation via intraspecific mortality should nevertheless be implemented in population models in order to avoid unrealistically high population densities. At least evidence for cannibalistic stages is already widespread in many animal groups, such as aquatic and terrestrial invertebrates, fish, birds, and mammals (Fox 1975; Polis 1981; Rosenheim and Schreiber 2022; Accolla et al. 2023), and therefore, the probability of regulation by density‐dependent mortality is also high for species that have not yet been investigated in this respect, especially if an intraspecific lethal interaction between defined stages of individuals seems plausible.

## 5 Conclusions

This study provided significant insights into the density‐dependent processes in *Chironomus riparius* populations, demonstrating that these mechanisms can lead to significant mortality at a population‐relevant scale. Our research offers a comprehensive analysis of the interplay between developmental delay due to food limitation and intraspecific mortality by interference competition, contributing to a deeper understanding of population regulation and dynamics in this important aquatic species.

By extending the standard laboratory experimental design for ecotoxicological studies to include reduced food and increased density levels, this study establishes a conceptual interface for the transferability of laboratory studies to populations under field conditions. This approach bridges the gap between controlled experiments and real‐world scenarios, enhancing the ecological relevance of laboratory‐based research.

A physiologically‐structured, individual‐based DEB model for *C. riparius* in its extended version for intraspecific interactions at the population level allows the realistic simulation of food‐dependent development and reproduction under the influence of direct interference and indirect exploitative competition. The generated data set we used for calibrating not only the density‐dependent mortality but also the half‐saturation constant for food uptake enables a more realistic modeling of population dynamics of *C. riparius* at both low and high larval densities. Using a modular modeling approach (Strauss et al. 2025), this model can be further extended by including new modules for the integration of complex environmental conditions and non‐density‐dependent processes, such as loss of adults and interspecific predation, to predict population dynamics under field conditions with increasing realism.

## Author Contributions

**Tido Strauss:** conceptualization (lead), data curation (lead), formal analysis (lead), investigation (lead), methodology (lead), project administration (equal), software (equal), supervision (lead), visualization (lead), writing – original draft (lead), writing – review and editing (lead). **Jana Gerhard:** data curation (supporting), formal analysis (supporting), software (equal). **Daniel Gerth:** data curation (supporting), investigation (supporting). **Josef Koch:** data curation (supporting), formal analysis (supporting), software (equal), writing – original draft (supporting). **Richard Ottermanns:** formal analysis (supporting), writing – original draft (supporting). **Maxime Vaugeois:** conceptualization (supporting), funding acquisition (equal), project administration (equal), supervision (supporting), writing – original draft (supporting). **Nika Galic:** conceptualization (supporting), funding acquisition (equal), project administration (equal), supervision (supporting), writing – original draft (supporting).

## Conflicts of Interest

The authors declare no conflicts of interest. However, Syngenta uses population models to assess the impact of chemicals on target and non‐target species in the context of pesticide regulation.

## Supporting information


**Data S1:** ece371949‐sup‐0001‐supinfo.docx.

**Data S2:** ece371949‐sup‐0002‐supinfo.docx.

## Data Availability

Experimental raw data and R codes used for the statistical analysis can be found on Dryad Digital Repository at: https://doi.org/10.5061/dryad.0rxwdbsd8.

## Appendix A

### Experimentally Derived Equation for the Density‐Dependent Mortality Rate [% d^−1^]

Figure A1 describes the dependence of the mortality rates on the initial larval density per 50 cm^2^ observed in the experiments. For this regression, the data of the food level of 0.25 mg larva^−1^ day^−1^, for which the highest number of larval densities was tested, was used (Regression equation: *y* = 0.124 x^0.4^ [% day^−1^], *R* ^2^ = 0.98).
