# Supplementary material for: What Is Regulating Chironomid Populations? The Influence of Food Supply and Interference Competition on Development and Mortality in Chironomus riparius
Source: Ecol Evol. 2025 Aug 13;15(8):e71949. doi: 10.1002/ece3.71949 (PMC12344276; doi:10.1002/ece3.71949)
Supplement: Supplementary file 1 — Data S1: ece371949‐sup‐0001‐supinfo.docx. [file ECE3-15-e71949-s001.docx]

**Supplemental Information for:**

**What is regulating chironomid populations? The influence of food supply and interference competition on development and mortality in *Chironomus riparius*.**

Tido Strauss, Jana Gerhard, Daniel Gerth, Josef Koch, Richard Ottermanns, Maxime Vaugeois, and Nika Galic

E-mail contact: [strauss@gaiac-eco.de](mailto:strauss@gaiac-eco.de)

**Table of Contents:**

[1 Test design overview 2](#_Toc197009989)

[2 Simulated impact of Monod kinetics on larval development 3](#_Toc197009990)

[3 IBM Chironomus model variability 5](#_Toc197009991)

[4 Oxygen measurements 7](#_Toc197009992)

[5 Larval dry weight measurements 8](#_Toc197009994)

[6 Sex-specific emerging success 9](#_Toc197009995)

[7 Measured and simulated total emergence over time 10](#_Toc197009996)

[8 Egg number (clutch size) simulations 12](#_Toc197009997)

[9 Statistical analysis of the experimental data 13](#_Toc197009998)

# Test design overview

The following table gives an overview of the test design of the experiments with regard to food level (mg per larva, test vessel and surface area) and larval density (per test vessel and surface area).

Table S 1: Overview on the general test design.

| **Larval density** | | | **Food level** | | | **Vessel surface area [cm^2^]** |
| --- | --- | --- | --- | --- | --- | --- |
| **Larvae per Vessel** | **Larvae per 50 cm^2^** | **Larvae per cm^2^** | **mg larva^-1^ day^-1^** | **mg vessel^-1^ day^-1^** | **g m^-2^ day^-1^** |  |
| 10 | 10 | 0.2 | 0.05 | 0.5 | 0.1 | 50 |
|  |  |  | 0.125 | 1.25 | 0.25 |  |
|  |  |  | 0.25 | 2.5 | 0.5 |  |
|  |  |  | 0.5 | 5 | 1 |  |
| 20 | 20 | 0.4 | 0.05 | 1 | 0.2 |  |
|  |  |  | 0.125 | 2.5 | 0.5 |  |
|  |  |  | 0.25 | 5 | 1 |  |
|  |  |  | 0.5 | 10 | 2 |  |
| 40 | 40 | 0.8 | 0.05 | 2 | 0.4 |  |
|  |  |  | 0.125 | 5 | 1 |  |
|  |  |  | 0.25 | 10 | 2 |  |
|  |  |  | 0.5 | 20 | 4 |  |
| 100 | 100 | 2 | 0.05 | 5 | 1 |  |
|  |  |  | 0.125 | 12.5 | 2.5 |  |
|  |  |  | 0.25 | 25 | 5 |  |
|  |  |  | 0.5 | 50 | 10 |  |
| 200 | 200 | 4 | 0.05 | 10 | 2 |  |
|  |  |  | 0.125 | 25 | 5 |  |
|  |  |  | 0.25 | 50 | 10 |  |
|  |  |  | 0.5 | 100 | 20 |  |
| 20 | 5.6 | 0.112 | 0.25 | 5 | 0.278 | 180 |
| 20 | 1.7 | 0.033 | 0.25 | 5 | 0.083 | 600 |
| 20 | 20 | 0.4 | (no food) | (no food) | (no food) | 50 |

# Simulated impact of Monod kinetics on larval development

In order to adequately model the influence of low amounts of available food per sediment area on larval food intake, a Monod kinetics with a half-saturation constant K_S_ was implemented, which reduces food intake at low food density. Exemplary simulations illustrate the impact of the coefficient K_S_ for ingestion on the emergence dynamics (see Figure S 1): Under the experimental conditions given in this study, changes in the half-saturation constant have a significant influence on the development rates in the scenarios with 600 cm^2^ aquaria. In contrast, even large changes of K_S_ in the range of 0 % to almost 300 % of the calibrated value have only a minor impact in the standard scenarios with 50 cm^2^ beakers. Without Monod kinetics (K_S_ = 0), there are no significant differences in the simulation results for larval development between large aquaria and small beakers with the same number of larvae and larvae-specific food supply, which contradicts the experimental data. This emphasises the need to introduce a half-saturation constant for food intake as a prerequisite for simulating scenarios at lower food and larval densities, but also the relevance of the aquarium experiments for calibrating the half-saturation constant K_S_. We assume that, with the simplifying assumptions we have made about food density dependence on feeding, we can adequately model growth inhibition at low food and larval densities at least in our experiments. Figure S 1 describes the impact of half-saturation constants on the emergence rate for two selected experimental scenarios of this study.


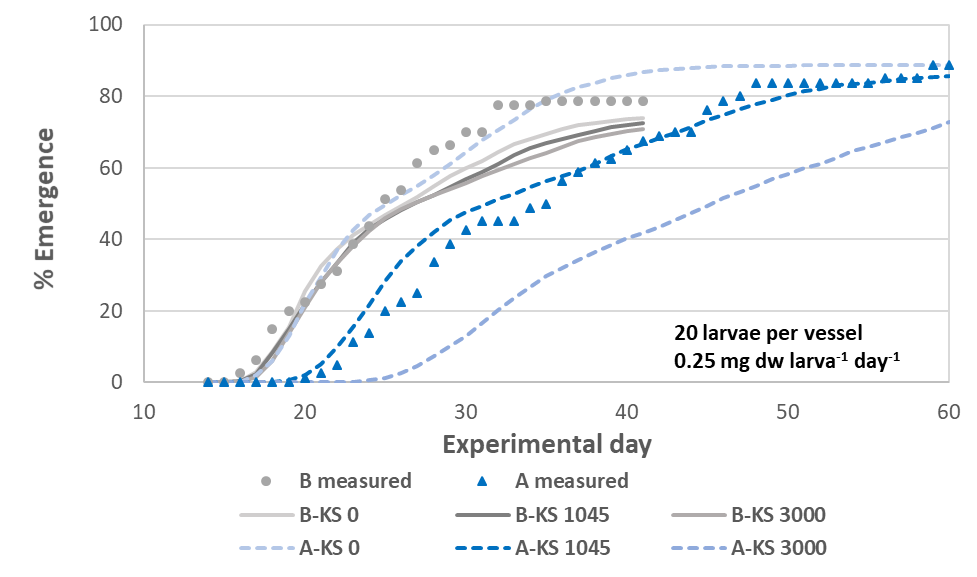


Figure S 1: Effect of the half-saturation constant K_S_ on development, exemplified for food quantity 0.25 mg larvae^-1^ day^-1^ and 20 larvae per vessel. B: 50 cm^-2^ beakers; A: 600 cm^-2^ aquaria. The measured values are labelled with symbols, the simulation results are given using different half-saturation constants K_S_ [J m^-2^] for beakers (solid lines) and aquaria (dashed lines).

# IBM Chironomus model variability

The following exemplary evaluation of the scatter between 20 Monte Carlo simulations per experimental scenario selected from this work is intended to give an impression of the robustness of the mean values obtained from individual simulations.

The evaluated endpoint is the percentage of cumulative emergence in relation to the number of initial larvae after 100 simulation days. The mean value and the minimum and maximum proportion of hatched adults for 20 Monte Carlo simulation runs are shown depending on the larval density [larvae m^-2^] and the food supply [kJ m^-2^ d^-1^].

The scattering of the total emergence is caused by the variable development speed of the individual larvae and the resulting probability of death depending on their development time. This random variation in cumulative occurrence per simulation run increases with decreasing initial larval density, leading to a maximum deviation from the mean of 0.6 % at 40,000 larvae m^-2^ and up to 6 % at 333 larvae m^-2^ (Figure S 2). A different food supply, on the other hand, has hardly any effect on the fluctuation of cumulative emergence within the respective food scenarios, with a stable maximum deviation from the mean around ± 2 % (Figure S 3).


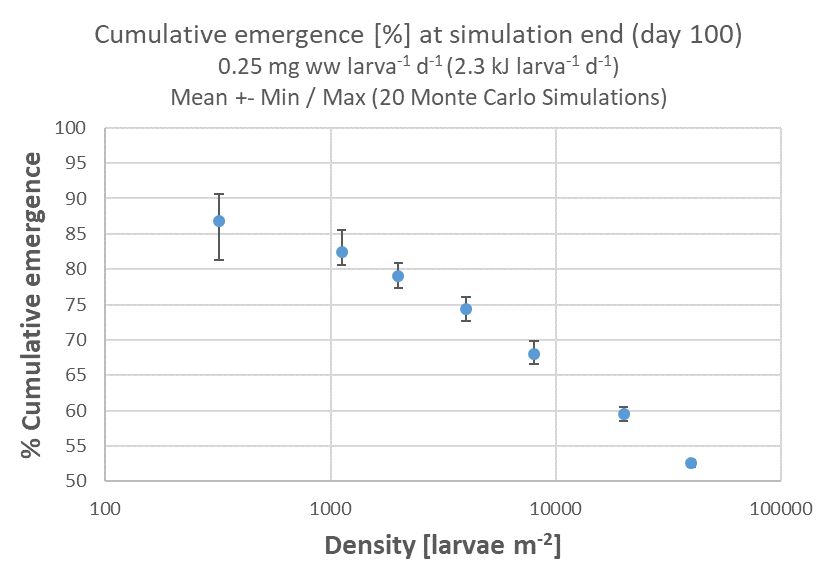


Figure S 2: % total emergence after 100 days of simulation. Mean ± min / max values for 20 Monte Carlo simulations per scenario. The seven larval densities between 333 and 40000 larvae m^-2^ in this figure correspond to 1.7, 5.6, 10, 20, 40, 100, and 200 larvae per 50 cm^2^ beaker at 0.25 mg food larva^-1^ day^-1^ (0.575 J larva^-1^ day^-1^).


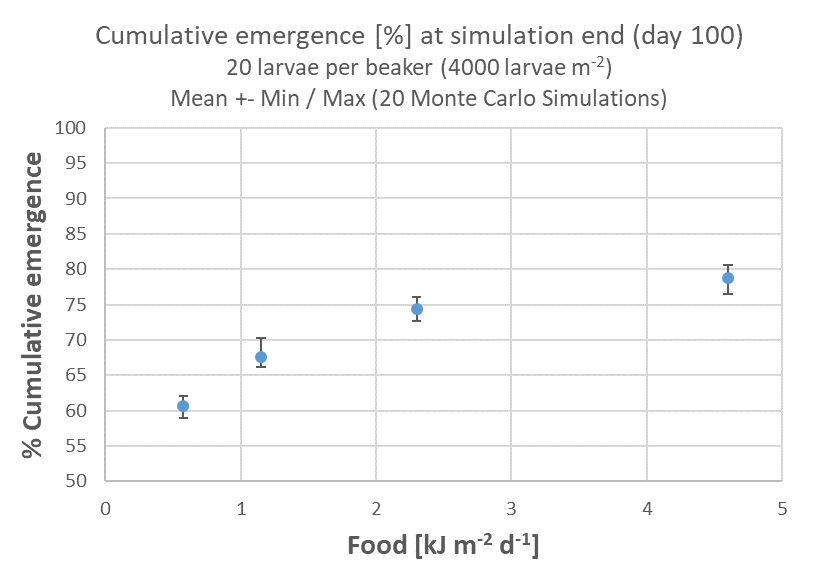


Figure S 3: % total emergence after 100 days of simulation. Mean ± min / max values for 20 Monte Carlo simulations per scenario. The four food levels correspond to 0.5, 0.25, 0.125, and 0.05 mg food larva^-1^ day^-1^ at 20 larvae per 50 cm^2^ beaker (4000 larvae m^-2^).

# Oxygen measurements

In the laboratory experiments, an oxygen concentration of 97.9 ± 5.7 % (mean ± SD) was measured in all treatments of the experiments only excluding the highest food supply scenario (0.5 mg larva d^-1^ at 200 larvae per 50 cm^2^ beaker), with single measured values between 60.8 and 104.4 % oxygen saturation. The percentage oxygen saturation in the highest food supply scenario was 43.1 ± 12.4 (mean ± SD) on days 14 and 19 (Figure S 4) and was thus clearly below 60 % saturation in most replicates.

Figure S 4: Oxygen saturation of all single replicates on days 14 and 19 (left) and on all other days of the experiment (right) versus total daily food supply per beaker (50 cm^2^).

# Larval dry weight measurements

All terminated replicates were examined for surviving larvae, which were subsequently dried and weighed together per replicate. The measurement results were divided by the number of larvae to calculate the mean larval dry weight per replicate. Living larvae at the end of the studied period could be found in the 0.05 mg larva^-1^ day^-1^ food level across all larval densities. Otherwise only in single two food level-larval density combinations and in the controls without food. The dry weights per surviving larvae are shown in Table S 2. The exemplarily determined dry weight of freshly hatched 1^st^ instar larvae in four samples with different numbers of pooled larvae per sample was 0.021 ± 0.005 mg dw larva^-1.^

Table S 2: Result overview of the dry-weight measurements of the surviving larvae after the experimental termination of the respective replicates.

| **Food level**  **[mg/ind]** | **Initial larval density** | **Number of measured replicates** | **Dry weight [mg] per surviving larvae** | |
| --- | --- | --- | --- | --- |
|  |  | **n** | **M** | **SD** |
| 0.05 | 10 | 3 | 0.79 | 0.28 |
| 0.05 | 20 | 2 | 1.18 | 0.51 |
| 0.05 | 40 | 4 | 1.45 | 0.50 |
| 0.05 | 100 | 4 | 0.67 | 0.25 |
| 0.05 | 200 | 4 | 1.32 | 0.46 |
| 0.125 | 10 | 1 | 2.09 | n.d. |
| 0.5 | 200 | 3 | 0.67 | 0.38 |
| no food | 20 | 3 | 0.066 | 0.005 |

# Sex-specific emerging success

Under all experimental conditions, the males hatched significantly earlier than the females. Figure S 5 shows the sum of emerging females and males per food level (sum of emergence across all 5 larval densities).


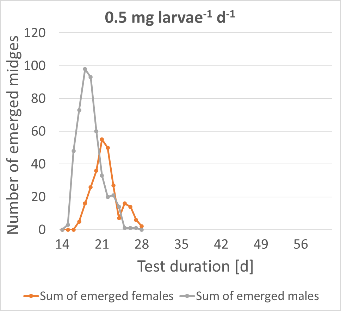

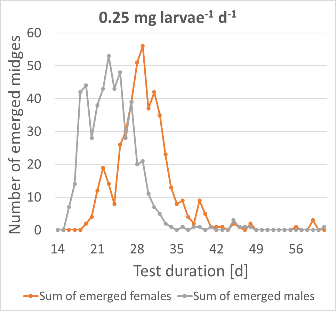

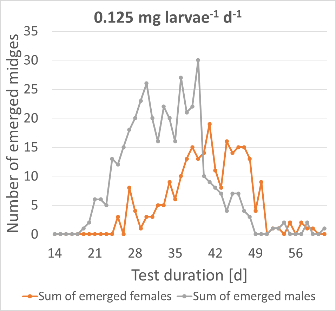

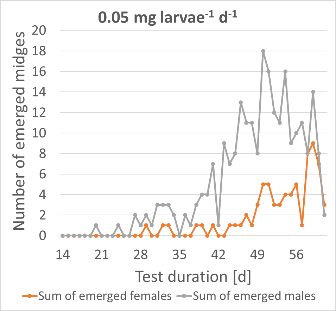


Figure S 5: Emergence of males (gray) and females (orange) across all larval densities within the different food levels. Decreasing food level from left to right: 0.5 - 0.25 - 0.125 - 0.05 mg larva^-1^day^-1^.

# Measured and simulated total emergence over time

The measured and simulated emergence (pooled across males and females) is shown in Figure S 6. Figure S 7 shows the emergence of animals that are still alive or have already emerged, taking mortality into account. A comparison of simulations for the highest feeding level (0.5 mg larvae^-1^ d^-1^) with simulations under unlimited conditions is shown in Figure S 8.

Figure S 6: Cumulative emergence success in measured data (mean values of replicates, symbols) and simulations (mean of 20 Monte Carlo simulations, simple lines). a) Different larval densities (10, 20, 40, 100, and 200 larvae per 50 cm^2^) per food level [mg food larvae^-1^ d^-1^]. b) Enlarged sediment area with 180 and 600 cm^2^ (20 initial larvae per aquarium at 0.25 mg food larvae^-1^ d^-1^.

Figure S 7: Cumulative emergence success in relation to the sum of hatched adults and surviving larvae with correction for dead animals. For legend see Figure S 6.

Figure S 8: Measured data (mean values of replicates, dots) and simulations (lines) of the highest food level scenario (0.5 mg larva^-1^ day^-1^) tested in the experiment. Simulations conducted with food addition of 0.5 mg larvae^-1^ d^-1^ (left) and without food limitation (right).

# Egg number (clutch size) simulations


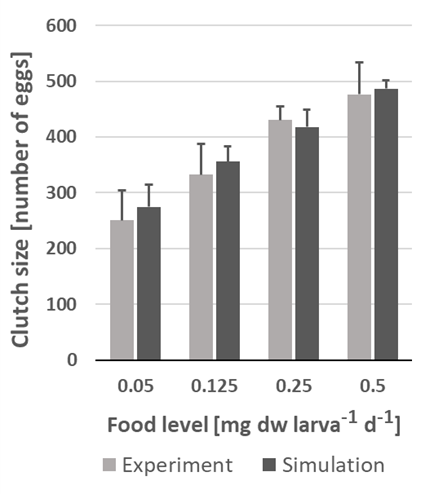


Figure S 9: Comparison of the 50 cm^2^ beaker experiments with simulations of the mean clutch size in the tested food-density combinations (M ± SD per food level, with n=5 different larval densities per food level).

# Statistical analysis of the experimental data

The main publication shows the correlations between the experimental findings for emergence rates, mortalities and reproduction as a function of food level and larval density. Table S 3 summarizes the corresponding statistical analysis with LM and GLM models.

Table S 4 lists the regression equations and determination coefficients for the trend model used in the main publication (Figure 3).

Table S 3: Overview on statistical evaluations of the experimental data (p-values of correlations).

|  | **Daily food per larva** | **Larval density** | **Comments** |
| --- | --- | --- | --- |
| **EmT_50_ [days] *^#^*** | 2.22e-14 *** | 0.42 | Only the (negative) food dependency is significant |
| **Mortality rate [%/d] *^##^*** | 0.355 | 2.07e-05 *** | Only the (positive) density dependency is significant |
| **Mortality at test end [%] *^##^*** | 5.84e-07 *** | 3.43e-06 *** | Both, the (positive) food dependency and the (negative) density dependency are significant |
| **Egg number per clutch *^###^*** | 1.07e-05 *** | 0.0477 * | The (positive) food dependency is highly significant, the (positive) density dependence is only weakly significant |

*#:* Multiple Spearman's rank correlation (Linear model, LM) without interaction

*##*: Generalized linear model (GLM) with beta distribution (betareg in R) without interaction

*###:* Generalized linear model (GLM) with quasi-Poisson distribution without interaction

Significance level: * p < 0.05; ** p < 0.01; *** p < 0.001

Table S 4: Overview of the regression coefficients and R^2^ of the regression trend model (y=ax^b^) for the experimental data in the main publication (regressions were calculated with SigmaPlot version 8.0).

|  | **Daily food per larva** | **Larval density** |
| --- | --- | --- |
| **EmT_50_ [days]** | 11.52x^-0.570^ (R^2^=0.89) | 30.48x^0.034^ (R^2^=0.008) |
| **Mortality rate [%/d]** | 0.44x^-0.186^ (R^2^=0.106) | 0.214x^0.29^ (R^2^=0.67) |
| **Mortality at test end [%]** | 14.48x^-0.419^ (R^2^=0.44) | 10.35x^0.307^ (R^2^=0.49) |
| **Egg number per clutch** | 593.04x^0.275^ (R^2^=0.77) | 285.79x^0.069^ (R^2^=0.08) |
