# Supplementary material for: What Is Regulating Chironomid Populations? The Influence of Food Supply and Interference Competition on Development and Mortality in Chironomus riparius
Source: Ecol Evol. 2025 Aug 13;15(8):e71949. doi: 10.1002/ece3.71949 (PMC12344276; doi:10.1002/ece3.71949)
Supplement: Supplementary file 2 — Data S2: ece371949‐sup‐0002‐supinfo.docx. [file ECE3-15-e71949-s002.docx]

**Description of the individual-based model**

**“IBM Chironomus population model”**

**for the aquatic midge *Chironomus riparius***

**-**

**ODD (Objects, Design concepts, and Details) protocol**

Version 2.1

**Laboratory application**

Authors

*Tido Strauss*

*Jana Gerhard
Josef Koch*

gaiac - Research Institute for Ecosystem Analysis and Assessment

An der Ölmühle 2, 52074 Aachen, Germany.

[strauss@gaiac-eco.de](mailto:strauss@gaiac-eco.de)

**13 December 2024**

Content

[1 Purpose 3](#_Toc187299475)

[2 Entities, state variables and scales 3](#_Toc187299476)

[3 Process overview and scheduling 6](#_Toc187299477)

[4 Design concepts 7](#_Toc187299478)

[4.1 Basic principles 7](#_Toc187299479)

[4.2 Model emergence 7](#_Toc187299480)

[4.3 Adaptation 7](#_Toc187299481)

[4.4 Sensing 7](#_Toc187299482)

[4.5 Interaction 7](#_Toc187299483)

[4.6 Stochasticity 8](#_Toc187299484)

[4.7 Observation 8](#_Toc187299485)

[5 Submodels 10](#_Toc187299486)

[5.1 Individual life history processes based on DEB theory 10](#_Toc187299487)

[5.2 Temperature dependency 19](#_Toc187299488)

[5.3 Model extensions for the population level 20](#_Toc187299489)

[5.3.1 Spatial scenario and larval movement 20](#_Toc187299490)

[5.3.2 Food resources 21](#_Toc187299491)

[5.3.3 Feeding 21](#_Toc187299492)

[5.3.4 Reproduction 22](#_Toc187299493)

[5.3.5 Survival 23](#_Toc187299494)

[5.3.6 Individual variability of feeding rate in Chironomus riparius 24](#_Toc187299495)

[6 Calibration routine 25](#_Toc187299496)

[7 Initialization 26](#_Toc187299497)

[*8* Case study-specific parameters for *C. riparius* 27](#_Toc187299498)

[9 Data input 29](#_Toc187299499)

[10 References 30](#_Toc187299500)

# Purpose

The implemented dynamic energy budget DEB module for *Chironomus riparius* enables the individual-based modelling of the development of newly hatched *Chironomus* larvae up to the emergence of adults as a function of food supply and temperature. The individual variability of the development is taken into account and the number of eggs per adult female is simulated. At the population level, the individuals compete for available food (exploitative competition) and react to the presence of conspecifics with intraspecific density-dependent mortality (interference competition).

With this modelling approach, different scenarios for laboratory experiments with *C. riparius* can be simulated, analysed and predicted.

This model description for the IBM Chironomus population model follows the ODD (Overview, Design concepts, and Details) protocol (Grimm et al., 2020).

# Entities, state variables and scales

The model comprises the following five **entities**: the environment, a spatial unit with grid cells, resources, individuals, and the population.

The entity of ‘**environment**’ refers to the overall environment and does not vary across space. It drives the behaviour and dynamics of all agents and their food resources on the grid cells based on external drivers such as water temperature.

The **spatial structure** of the environmental scenario is realized through a structural and functional parametrisation of individual **grid** **cells**. The size and position of the cells is defined by a scenario specific grid map, which is read in from an external text file. Each cell is embedded in the landscape grid and characterized by its X/Y coordinates. A grid cell is further characterized by its environmental factors (i.e. microhabitat type and food resource content), which are determined by the study site or hypothetical ecological scenarios.

The entity ‘**resources**’ characterizes the specific parameters and functions that control the behaviour of the food resource for the chironomids.

The **individual** object includes all state variables required to compute the life cycle of an individual chironomid. The metabolic processes of the individuals are mainly based on the DEB-Theory. A detailed overview of the DEB-related parameters and the DEB model can be found in Section 5.1.

The entity ‘**population**’ has a kind of bookkeeping function in the model. It provides information of the abundance and spatial distribution of all modelled individuals, including hatched adults, new eggs, and dead individuals (for details see Section 4.7).

In its current state, the main chironomid population level endpoints over the simulation period provided by the model are: i) The total larvae density and the densities of their developmental stages [# m^-2^], ii) the sex-specific number of emerged adults, iii) the number of eggs deposited in the modelled system, and iv) the individual causes of death (e.g. by starvation, density-dependent mortality, etc.). Further details on the model output can be found in Section 4.7.

The biological model for the individual organisms follows the five **state variables** (age, size in terms of structural length, energy reserve, reproduction buffer, and maturity. In addition, the amount of food available in the environment is also modelled as a state variable (see Table 2.1).

Table 2.1: Environmental and individual-specific state variables of the IBM Chironomus population model.

| **Variable** | **Symbol** | **Unit** |
| --- | --- | --- |
| **Environment** |  |  |
| Food density | *X* | [J m^-2^] |
| **Individual** |  |  |
| Age since birth | *age* | [d] |
| Scaled reserve | $\boldsymbol{U}_{\boldsymbol{E}}$ | [d cm²] |
| Structural length | $\boldsymbol{L}$ | [cm] |
| Scaled maturity | $\boldsymbol{U}_{\boldsymbol{H}}$ | [d cm²] |
| Scaled reproduction buffer | $\boldsymbol{U}_{\boldsymbol{R}}$ | [d cm²] |

**Temporal scales:** The model is designed for simulations lasting from several weeks to several years. We used discrete time steps in the implementation of the DEB model. During a simulation, the calculation of the individual feeding rates and the update of the consumed resources will occur at hourly time steps, all other physiological processes, such as growth and development, emergence of the adults, mortality, and movement, are calculated in daily time steps.

Spatial scales: Spatial scenarios between 0.5 m^2^ and up to around 1000 m^2^ can be read in, whereby the computer memory requirements and computing time increase significantly with the total number of animals modelled (for details see Section 5.3.1).

# Process overview and scheduling

In this IBM Chironomus population model, an IBM approach is used to simulate the life cycle of each individual in an aquatic environment.

The life history of individuals depends on the actual food intake, and the ambient water temperature. They grow, reproduce and eventually die, depending on aging, predation, or starvation (e.g. in competition situations).

Within each 24 hour period, the following processes are executed in sequence:

At the beginning of a day, the forcing variables such as water temperature are read in from data files. The addition of food is then calculated.

Subsequently, each animal runs through the physiological dynamic-energy-budget (DEB) cycle. This includes a one-hour loop for the food intake of all larvae and the subsequent updating of the food resource.

After 24 cycles of hourly calculations for food consumption of the larvae, all physiological DEB state variables of each individual are updated, and the number of individuals that died on this day due to starvation or aging are counted.

The density dependent mortality of the larvae is then calculated and assigned to individuals. The probability is calculated with which surviving larvae that have not yet pupated move to another, adjacent grid cell.

All larvae and emerged adults that have died on this day are counted and then removed from the population. An up-to-date count of all living life stages is then carried out.

# Design concepts

# Basic principles

The purpose of the IBM Chironomus population model is the temporal and spatial simulation of the individual-based dynamics of chironomid populations under different environmental conditions and population densities.

The entire life cycle of individuals is simulated on the basis of DEB theory. A detailed description of all DEB processes can be found in Section 5.1.

# Model emergence

Individual life cycle state variables are based on the species-specific life history traits and the metabolic organisation of the modelled organisms. Chironomid population dynamics, including total abundance and developmental stage composition, emerge from the development of each individual chironomid dependent on environmental conditions and density-dependent interactions.

# Adaptation

The larval movement probability within the spatial set up (between grid cells) is influenced by their internal energy status and thus indirectly by availability of food.

# Sensing

The parameters sensed by the individual chironomid larvae are the water temperature, the larval densities and food densities of the respective grid cell on which they reside.

# Interaction

Intraspecific density-dependent mortality (interference competition) is the only direct interaction between individuals, based on larval density.

Indirect effects between individuals occur through the sharing of food resources (exploitative competition).

# Stochasticity

The nature of the IBM Chironomid population model is stochastic. This section lists the stochastically influenced parameters and processes.

When initialising the model, the initial population can have randomised and therefore different size structures for each simulation in a previously specified size range, and the initial spatial distribution of individuals across the grid cells is also random (see Section 7).

Individuals differ in some parameters from birth. Sex is determined randomly at birth with a 50% probability. To account for the natural variability of individual physiology within a population, the individual maximum assimilation rate of an animal is calculated at birth using a defined probability distribution (see Section 5.3.6).

The susceptibility of each individual larva within the population to be killed as a result of density-dependent mortality (see Section 5.3.5), and the susceptibility of each emerged adult to be lost as a result of a daily calculated adult loss rate *mr* (see Section 5.3.4), is determined by an individual random number between 0 and 1, which is renewed daily. If the individual random number exceeds the calculated process-specific probabilities in the population, the individual is not affected.

Another source of stochasticity comes from the movement of the larvae between grid cells. In this model, both the probability of movement as well as the direction of movement of the individuals are stochastic (see Section 5.3.1).

Each day, a separate value is randomly drawn from a uniform distribution between 0 and 1 for each individual and each process-specific probability number.

Finally, the calculation order for the individuals in a population is chosen at random on each simulation day.

# Observation

During the simulations, all life stages of living individuals are summed up daily (see Table 4.7.1).

In addition, the number of adult midges that have emerged, the mean number of eggs per female, the number of animals that have died (stating the cause of death), and the amount of food available per area are saved daily.

Table 4.7.1: Implemented model outputs.

| **Environment** | **Unit** |
| --- | --- |
| Water temperature* | [°C] |
| Food density | [J m^-2^] |
| **Population** | **Unit** |
| Population size (total number of individuals, all stages) | [# m^-2^] |
| Eggs | [# m^-2^] |
| New laid eggs per day | [# m^-2^ d^-1^] |
| Daily average of eggs per female | [# female^-1^] |
| Juveniles (still without energy investment in reproduction) | [# m^-2^] |
| Adults (with energy investment in reproduction) | [# m^-2^] |
| Pupae | [# m^-2^] |
| Emerged adults per day | [# m^-2^ d^-1^] |
| Density dependent mortality | [# m^-2^ d^-1^] |
| Death by starvation | [# m^-2^ d^-1^] |
| Death by aging | [# m^-2^ d^-1^] |

*: Water temperature as unchanged input data

By default, the simulation results are saved daily as mean values per m^2^ for the entire simulation area but can also be read out specifically for individual grid cells if required.

In the case of Monte Carlo simulations, the individual results of all simulation runs are saved per parameter in a data file and are available for subsequent calculations and statistical analyses.

# Submodels

# Individual life history processes based on DEB theory

This chapter presents several DEB model versions for different species-specific life histories that are implemented in the IBM Chironomus population model and can be selected on a species-specific basis.

**Please note:** For the dipteran species *Chironomus riparius*, the **DEB model *hax*** is used (see below).

Nomenclature notes for variable abbreviations in DEB:

- “per unit of structural volume” is indicated by square brackets [ ],
- “per unit of structural surface area” is indicated by curly brackets { },
- Rates (i.e., per unit of time) have dots.

Life histories of individual organisms are modelled based on the Dynamic Energy Budget (DEB) theory (Kooijman, 2010). DEB models describe individual life-history processes based on energy fluxes: Organisms assimilate resources from their environment and subsequently allocate energy through a reserve compartment to maintenance, somatic growth and the reproduction system. In the reproduction system, maturity is built up over time (also increasing the running costs for maturity maintenance) until the life-history event of puberty is reached. After this, maturation ends and reproduction begins (Figure 5.1.1).

Variables and parameters are listed in Table 5.1.1 and Table 5.1.2 respectively whereas model equations are given in Table 5.1.3.

Depending on the modelled species, we either use the **standard DEB model (*std*)** or one of the model extensions ***abj*** (metabolic acceleration from birth until the life-history event of metamorphosis, denoted by the letter *j*), ***hep*** (like *abj* but offspring is released only once at the life-history event emergence instead of regular brood intervals) and ***hax*** (model for holometabolic insects with a long larval stage that includes a pupa stage).

The ***std*-model** applies to most animal species without larval phases since it assumes isomorphy throughout the full life cycle. The ***abj*–model** applies to most species with a larval phase and is a one-parameter extension of std-model. The model is like the std-model, but it includes a type Μ acceleration between birth $E_{H}^{b}$ (or $U_{H}^{b}$ in the scaled model) and metamorphosis $E_{H}^{j}$ (or $U_{H}^{j}$ in the scaled model). Metamorphosis is before puberty, which might or might not correspond with changes in morphology. During metabolic acceleration, $\{\dot{p}_{Am}\} = \{\dot{p}_{Am}^{b}\}L/L_{b}$ and $\dot{v} =\dot{v}^{b} L/L_{b}$, where $\{\dot{p}_{Am}^{b}\}$ and $\dot{v}^{b}$ refer to the values at birth. At metamorphosis, acceleration ceases: $\{\dot{p}_{Am}\} = \{\dot{p}_{Am}^{b}\}s_{M}$ and $\dot{v} =\dot{v}^{b} s_{M}$, with acceleration factor $s_{M}= L_{j}/L_{b}$.

The ***hep*-model** is a model for hemi-metabolic insects, such as ephemeropterans, odonata and possibly other insect groups. It includes eggs, larvae and (sub)imagos as morphological life stages and embryos, juveniles, adults and imagos as functional stages. The embryo development follows the *std*-model but between birth $E_{H}^{b}$ and puberty $E_{H}^{p}$ the model assumes type M acceleration. The transformation to subimago occurs when the reproduction buffer exceeds a threshold $\left[ E_{R}^{j} \right]$.

Like abj and hep, the ***hax*-model** also deviates from the std model in that the animal experiences a phase of metabolic acceleration between the birth and puberty. While no further life history events occur after puberty in the std model (except for regular releases of eggs/neonates), hax animals undergo the additional events of pupation and emergence. Pupation – according to the documented hax model assumptions at the time of writing (Kooijman, 2010, update 2023-05-05) occurs as soon as a critical threshold of reproduction buffer density $\left[ E_{R}^{j} \right]$ is reached. At this point, all body structure is transformed into reserve energy, and maturity is reset to zero. The pupa does not feed externally but draws from its reserve, much like the embryo (i.e., pre-birth) stage of the DEB models. Emergence is triggered by a threshold of maturity. The imagos, who still do not feed, finally transform their reproduction buffer into a single egg mass and are assumed to die shortly after.

Figure 5.1.1: Schematic representation of the standard DEB model and exemplary physiological modes of action (see below); modified from Álvarez et al. (2006).

Table 5.1.1: Variables and intermediate outputs for modelling life histories.

| Symbol | Parameter description | Unit |
| --- | --- | --- |
| $\boldsymbol{S}_{\boldsymbol{C}}$ | scaled mobilisation flux of reserves | [${cm}^{2}$] |
| $\boldsymbol{e}$ | scaled reserve density | $[-]$ |
| $\boldsymbol{L}$ | structural length | [$cm$] |
| $\boldsymbol{l}$ | scaled structural length | $[-]$ |
| ${\dot{\boldsymbol{p}}}_{\boldsymbol{X}}$ | ingestion rate | [$Jd^{-1}$] |
| ${\dot{\boldsymbol{p}}}_{\boldsymbol{A}}$ | assimilation rate | [$Jd^{-1}$] |
| $\boldsymbol{f}$ | scaled functional response | $[-]$ |
| $\dot{\boldsymbol{R}}$ | reproduction rate | [$\#d^{-1}$] |
| $\boldsymbol{U}_{\boldsymbol{H}}$ | scaled maturity | [$d cm^{2}$] |
| $\boldsymbol{U}_{\boldsymbol{R}}$ | scaled energy in reproduction buffer | [$d cm^{2}$] |
| $\boldsymbol{U}_{\boldsymbol{E}}$ | scaled reserve | [$d cm^{2}$] |
| $\ddot{\boldsymbol{q}}$ | aging acceleration | [$d^{-2}$] |
| $\dot{\boldsymbol{h}}$ | hazard rate | $[d^{-1}]$ |
| $\boldsymbol{S}$ | survival probability | $[d^{-1}]$ |

Table 5.1.2: Parameters for modelling life histories.

| Symbol | Parameter description | Unit |
| --- | --- | --- |
| $\boldsymbol{g}$ | energy investment ratio | $[-]$ |
| ${\dot{\boldsymbol{k}}}_{\boldsymbol{M}}$ | somatic maintenance rate coefficient | [$d^{-1}$] |
| $\dot{\boldsymbol{v}}$ | energy conductance | [$d^{-1}$] |
| $\boldsymbol{E}_{\boldsymbol{R}}$ | energy in the reproduction buffer | [$J$] |
| $\left[ \boldsymbol{E}_{\boldsymbol{R}} \right]$ | reproduction buffer density | [${J cm}^{-3}$] |
| $\left[ \boldsymbol{E}_{\boldsymbol{G}} \right]$ | volume-specific costs of structure | [${J cm}^{-3}$] |
| $\boldsymbol{\kappa}$ | fraction of mobilised reserve allocated to soma | $[-]$ |
| $\boldsymbol{\kappa}_{\boldsymbol{R}}$ | fraction of reproduction energy fixed in eggs | $[-]$ |
| $\boldsymbol{\kappa}_{\boldsymbol{G}}$ | fraction of growth energy fixed in structure | $[-]$ |
| $\boldsymbol{\kappa}_{\mathbf{V}}$ | fraction of energy in mobilised larval structure fixed in pupal reserve | $[-]$ |
| ${\dot{\boldsymbol{k}}}_{\boldsymbol{J}}$ | maturity maintenance rate coefficient | [$d^{-1}$] |
| $\left\{ {\dot{\boldsymbol{p}}}_{\boldsymbol{Am}} \right\}$ | surface-area-specific maximum assimilation rate | $[J {cm}^{-2} d^{-1}]$ |
| $\left\{ {\dot{\boldsymbol{p}}}_{\boldsymbol{Xm}} \right\}$ | surface-area-specific maximum specific feeding rate | $[J {cm}^{-2} d^{-1}]$ |
| $\boldsymbol{\kappa}_{\boldsymbol{X}}$ | fraction of food energy fixed in reserve (assimilation efficiency) | $[-]$ |
| $\boldsymbol{U}_{\boldsymbol{H}}^{\boldsymbol{b}}$ | scaled maturity at birth | $[d cm^{2}]$ |
| $\boldsymbol{U}_{\boldsymbol{H}}^{\boldsymbol{j}}$ | scaled maturity at metamorphosis | $[d cm^{2}]$ |
| $\boldsymbol{U}_{\boldsymbol{H}}^{\boldsymbol{p}}$ | scaled maturity at puberty | $[d cm^{2}]$ |
| $\boldsymbol{U}_{\boldsymbol{H}}^{\boldsymbol{e}}$ | scaled maturity at emergence | $[d cm^{2}]$ |
| $\boldsymbol{u}_{\boldsymbol{E}}^{\boldsymbol{0}}$ | initial scaled reserve | $[-]$ |
| ${\ddot{\boldsymbol{h}}}_{\boldsymbol{a}}$ | Weibull aging acceleration | [$d^{-2}$] |
| $\boldsymbol{s}_{\boldsymbol{G}}$ | Gompertz stress coefficient | $[-]$ |
| $\boldsymbol{s}_{\boldsymbol{M}}$ | acceleration factor | $[-]$ |
| $\boldsymbol{L}_{\boldsymbol{b}}$ | structural length at birth | $[cm]$ |
| $\left[ \boldsymbol{E}_{\boldsymbol{R}}^{\boldsymbol{j}} \right]$ | Reproduction buffer density at emergence | $[J {cm}^{-3}]$ |

In this model, the acquisition of resources from the environment depends on the environmental food density $X$ [J m^-2^]. We use a functional response type II (Holling, 1959) for modelling food ingestion, with a half-saturation constant $K_{S}$ for food uptake [J m^-2^]. Many modifications to the type II function have been suggested (Jeschke et al., 2002); a general interpretation for the ingestion rate $\dot{p}_{X}$ can be based on a maximum ingestion rate $\left\{ \dot{p}_{Xm} \right\}$: the ingestion rate $\dot{p}_{X}$ is determined by the squared structural length $L^{2}$ of an individual times the surface-area-specific maximum ingesting rate $\left\{ \dot{p}_{Xm} \right\}$. The theoretical ingestion rate is calculated according to eq. 1. However, since the model takes into account additional feedback from the environment, the realized ingestion rate may differ (e.g., if less food is available than would theoretically be eaten). Therefore, a more detailed description of the submodel for feeding is provided in Section 5.3.3.

The ingested food is taken up into the body with some efficiency $\kappa_{X}$, which is usually assumed to be constant for a particular type of resource (van der Meer, 2006) and is independent of body size (Hendriks, 1999), to derive the assimilation rate $\dot{p}_{A}$ (eq. 2). The DEB scheme makes use of a scaled functional response $f$ that can exhibit values between 0 and 1. $f$ can be computed by dividing the realized assimilation rate by the maximum assimilation rate (i.e., the surface-area-specific maximum assimilation rate times the structural surface area) (eq. 3).

It should be noted that for further energy allocations within the organism, we use the scaled DEB model. By dividing all state variables and fluxes of energy (which can also be expressed in units of mass) by the surface-area-specific maximum assimilation rate, the units of energy or mass are removed from the model.

Assimilated energy is allocated to a reserve compartment. The scaled amount of reserve $U_{E}$ depends on the mobilization flux $S_{C}$ and with the onset of feeding at birth, also on the assimilation flux (eq. 4). For embryos, $\dot{p}_{X}$, $\dot{p}_{A}$, and $f$ are 0. Under growth conditions, the mobilization flux $S_{C}$ depends on the structural length $L$, the energy investment ratio $g$, the scaled reserve density $e$, the energy conductance $\dot{v}$ and the rate coefficient for somatic maintenance costs $\dot{k}_{M}$ (eq. 5); the scaled reserve density $e$ is given by eq. 6. Growth conditions are true if the scaled reserve density exceeds the scaled length ($l$) (eq. 7). At conditions of low resource supply, the scaled reserve density may drop below the non-growth boundary, and the animal is considered to be starving. There are several DEB starvation options available from the literature. For an overview see, e.g., (Jager and Zimmer, 2012). Often it is reasonable to assume that upon starvation, reserve is used to pay somatic maintenance costs only. If assuming that the animal does not shrink in structure (weight loss due to reserve utilization is still possible) the mobilization flux is given by eq. 8, where $\kappa$ is the reserve fraction that is allocated to the soma. An alternative assumption is that under starvation the organism shrinks in terms of structure, which is transformed back into reserve energy, to provide enough energy to sustain the reserve mobilization flux without any alterations to the mobilization rate (eq. 5). This assumption was proposed also for *C. riparius* by (Augustine and Gergs, 2019). Note that there is an energetic loss associated with the retransformation of structure into energy (assumed equal to the energetic loss during build-up of structure $\kappa_{G}$, which is included in the costs for growth parameter $\left[ E_{G} \right]$).

The net result of reserve allocation to soma $\kappa$ minus the somatic maintenance costs is used for growth, whereas the remaining fraction $(1-\kappa)$ minus the maturity maintenance costs is used for maturation and reproduction. The increase in structural length $L$ is given by eq. 9, or, alternatively, if the amount of reserve does not allow the organism to growth, i.e., $e\leq l$, and if structural shrinking is not assumed, then $dL/dt=0$. For some species (e.g., abj, hep, hax models), we apply the concept of metabolic acceleration to account for the initial slow growth observed during their ontogeny. For examples and a discussion of concepts see (Kooijman, 2014). Here, we assume that individuals accelerate their assimilation rate at some stage during their development using the acceleration factor $s_{M}$ applied to the parameters $\left\{ \dot{p}_{Xm} \right\}$, $\left\{ \dot{p}_{Am} \right\}$, and $\dot{v}$. For a brief derivation see (Zimmer et al., 2014). $s_{M}$ is given by the ratio of the structural length of the organism divided by its structural length at birth ($L_{b})$. Once the scaled maturity at metamorphosis ($U_{H}^{p}$) is reached, $s_{M}$ does not further increase and remains constant (structural length at metamorphosis divided by structural length at birth).

The change in scaled maturity $U_{H}$ depends on the fraction $(1-\kappa)$ of the mobilization flux $S_{C}$ minus the maturity maintenance costs (eq. 10), where $\dot{k}_{J}$ is the maturity maintenance rate. Fixed maturity levels mark the onset of feeding ($U_{H}^{b}$), the end of metabolic acceleration ($U_{H}^{j}$), and the switch from maturation to ($U_{H}^{p}$). Note that in the model types hep and hax, the latter two events coincide, and in literature are linked to $U_{H}^{p}$ with no need for the additional parameter $U_{H}^{j}$. After reaching puberty ($U_{H}^{p}$), maturation stops, and the mobilized reserve is allocated to the reproduction buffer (eq. 11) instead. For some species, the reproduction buffer is continuously converted into a reproduction rate $\dot{R}$ with a fixed reproduction efficiency $\kappa_{R}$, depending on the energetic costs for producing a single egg (eq. 12). For most holometabolous insects, including *C. riparius*, the reproduction buffer is accumulated until emergence occurs, and is then converted into eggs at once, again with a reproduction efficiency $\kappa_{R}$.

Aging is a major cause of natural death in animal species (additional mortality causes are described in Section 5.3.5). Aging acceleration summarizes the production of damage producing compounds that are accumulated and copied at rates proportional to the rate of energy mobilization. Aging comes with two extra parameters, the aging stress coefficient $s_{G}$ and the aging acceleration constant $\ddot{h}_{a}$(eq. 13); the resulting change in cumulative hazard rate $\dot{h}$ due to ageing is given by eq. 14. The survival probability $S$ resulting from aging is given by eq. 15. In this model implementation, the aging formulae were implemented as being optional. For *C. riparius*, aging-related mortality was disregarded since in holometabolous insects age-mortality typically occurs only after adult emergence, the state at which the imagos leave the aquatic system, and thus are also no longer modelled explicitly in this model.

The pupa stage of the hax model requires some additional considerations: Normally, as soon as the reproduction buffer density $\left[ E_{R} \right]=\frac{E_{R}}{V}= \frac{U_{R}*\left\{ \dot{p}_{Am} \right\}}{V}$ reaches a threshold $\left[ E_{R}^{j} \right]$, it is assumed that the pupation is initiated. However, based on the insights and elaborations provided by Koch et al. (2024, Section S5), in this model we used a slightly modified pupation assumption using a critical threshold of the ratio between the reproduction buffer $E_{R}$ ($U_{R}*\left\{ \dot{p}_{Am} \right\}$) and the structural length $L$ rather than the ratio $E_{R}$/$V$ described in the DEB literature. At the onset of pupation, all structure is transformed back into reserve energy with conversion efficiency $\kappa_{V}$. Furthermore, the scaled maturity $U_{E}$ is reset to zero and the metabolic acceleration factor is now permanently set to one. Pupal development follows the same rules as an embryo (i.e., pre-birth): no assimilation, but reserve mobilization, maturation and growth, according to the equations provided in Table 5.1.3. Finally, as soon as the scaled maturity $U_{E}$ of the pupa reaches the scaled maturity threshold for emergence $U_{H}^{e}$, the individual emerges as an imago and the reproduction buffer energy is transformed into eggs with efficiency $\kappa_{R}$. Imagos are then removed from the model because they are no longer relevant to the aquatic system once their eggs have been deposited.

To address the influence of different temperatures on the life history processes, we multiplied the Arrhenius temperature correction factor to all metabolic and physiological rates ($\dot{p}_{X}$*,* $\dot{p}_{A}$*,* $\frac{{dU}_{H}}{dt}$*,* $\frac{{dU}_{E}}{dt}$*,* $\frac{{dU}_{R}}{dt}$ $\frac{dL}{dt}$*,* $\frac{d\dot{h}}{dt}$*,* $\frac{d\ddot{q}}{dt}$*,* $\dot{h}$).

As stated above, all rates and fluxes except the ingestion rate (which is computed hourly and then summed up over 24 h to calculate the realized ingestion rate for a full day; see Section 5.3.3) were computed in discrete time steps of 1d.

**Table 5.1.3:** **Model formulations for representing energy acquisition and use in individual organisms.**

| **Equations** | **No.** |
| --- | --- |
| *Feeding and assimilation processes* | |
| $\dot{p}_{X}={\{\dot{p}}_{Xm}\}*s_{M}*L^{2}* \frac{X}{K_{S}+X}$ | eq. 1 |
| $\dot{p}_{A}=\kappa_{X}*\dot{p}_{X}$ | eq. 2 |
| $f=\dot{p}_{A}*\left( {\{\dot{p}}_{Am}\}*s_{M}*L^{2} \right)^{-1}$ | eq. 3 |
| *Reserve dynamics* | |
| $\frac{{dU}_{E}}{dt}=\frac{\dot{p}_{A}}{{\{\dot{p}}_{Am}\}}-S_{C}=s_{M}*L^{2}*f-S_{C}$ | eq. 4 |
| $S_{C}=L^{2}*g*e*(1+\dot{k}_{M}*L*{(\dot{v}*s_{M})}^{-1} ){(g+e)}^{-1}*s_{M}$ | eq. 5 |
| $e=\dot{v}*U_{E}*L^{-3}$ | eq. 6 |
| $l=L*\dot{k}_{M}*g*\left( \dot{v}*s_{M} \right)^{-1}$ | eq. 7 |
| $S_{C}=\kappa*\dot{k}_{M}*g*L^{3}* \dot{v}^{-1}$ (option for starvation condition) | eq. 8 |
| *Growth* | |
| $\frac{dL}{dt}=\frac{1}{3}L^{-2}* (\dot{v}*g^{-1}*S_{C} - \dot{k}_{M}*L^{3})$ | eq. 9 |
| *Maturation and reproduction* | |
| $\frac{{dU}_{H}}{dt}=\left( 1-\kappa\right)*Sc-\dot{k}_{J}*U_{H}$ for $U_{H}<U_{H}^{p}$ else $\frac{{dU}_{H}}{dt}=0$ | eq. 10 |
| $\frac{{dU}_{R}}{dt}=\left( 1-\kappa\right)*Sc-\dot{k}_{J}*U_{H}^{p}$ for $U_{H}\geq U_{H}^{p}$ else $\frac{{dU}_{R}}{dt}=0$ | eq. 11 |
| $\dot{R}=\kappa_{R}*U_{R}*{u_{E}^{0}}^{-1}$ | eq. 12 |
| *Survival due to aging and starvation* | |
| $\frac{d\ddot{q}}{dt}= \left( \ddot{q}*L^{3}* \left( \dot{v} g^{-1} {\dot{k}_{M}}^{-1} \right)^{-3}*s_{G}+ \ddot{h}_{a} \right)*e*\left( \dot{v}* L^{-1}- 3 \frac{dL}{dt}* L^{-1} \right)- 3 \frac{dL}{dt}* L^{-1}* \ddot{q}$ | eq. 13 |
| $\frac{d\dot{h}}{dt}=\ddot{q} - 3*\frac{dL}{dt} L^{-1}*\dot{h}$ | eq. 14 |
| $S=exp(-\dot{h}(t))$ eq. 15 | |

# Temperature dependency

To account for temperature dependency of the modelled processes, a temperature correction factor $TC$ (−) can be used, which is consistently applied to all physiological rates:

|  | $TC=\left\{ \begin{aligned} \exp\left( \frac{T_{A}}{T_{REF}}-\frac{T_{A}}{T} \right), &T<T_{REF} \\ s_{H}\exp\left( \frac{T_{A}}{T_{REF}}-\frac{T_{A}}{T} \right), &T\geq T_{REF} \\ s_{L}\exp\left( \frac{T_{A}}{T_{REF}}- \frac{T_{A}}{T} \right), &T\leq T_{REF} \end{aligned} \right.$ |  |
| --- | --- | --- |

with the upper boundary correction $s_{H}$ (−):

|  | $s_{H}=\frac{1+exp \left( \frac{T_{AH}}{T_{H}}-\frac{T_{AH}}{T_{REF}} \right)}{1+exp \left( \frac{T_{AH}}{T_{H}}-\frac{T_{AH}}{T} \right)}$ |  |
| --- | --- | --- |

and the lower boundary correction $s_{L}$ (−):

|  | $s_{L}=\frac{1+exp \left( \frac{T_{AL}}{T_{REF}}-\frac{T_{AL}}{T_{L}} \right)}{1+exp \left( \frac{T_{AL}}{T}-\frac{T_{AL}}{T_{L}} \right)}$ |  |
| --- | --- | --- |

Within the reference temperature $T_{REF}$ (K), the temperature dependency is described by only two parameters: $T_{REF}$ and the Arrhenius temperature $T_{A}$ (K). At and above $T_{REF}$, two additional parameters, i.e., the upper boundary temperature $T_{H}$ (K) and the Arrhenius temperature for the upper boundary $T_{AH}$ (K), are used to model a flattening of the temperature curve where physiological tolerance is exceeded. At and below $T_{REF}$, the lower boundary temperature $T_{L}$ (K) and the Arrhenius temperature for the lower boundary $T_{AL}$ (K), are used to model a delay of the temperature curve where physiological tolerance falls below the optimal temperature range.

Depending on the simulated temperature range and the data available for parameterising $T_{AH}$ and $T_{AL}$, temperature corrections can also be applied specifically for high or low temperature ranges.

# Model extensions for the population level

This section describes additional adaptations of the model for the simulation of *Chironomus riparius* populations that go beyond the pure DEB model specifications for the laboratory experiments of Koch et al. (2014).

These include the basic spatial scenario and larval movement (Section 5.3.1), an module for food (Section 5.3.2) and food uptake (Section 5.3.3), the modelling of reproduction (Section 5.3.4) as well as the adult loss rates and causes of larval death (Section 5.3.5). Finally, an additional introduction of individual variability for the larval ingestion rate is described (Section 5.3.6).

# Spatial scenario and larval movement

The simulated ecosystem is represented as a 2D grid map. We used a square area with a total size of 0.5 m², where the entire area was defined as benthic habitat. If the conditions in a grid cell are not optimal, larvae can also migrate into other cells looking for better conditions. In the current model implementation, the probability to stay can generally be calculated from the preferences of a macroinvertebrate for a substrate type, the water depth, for a water flow velocity class as well as the energy content of an individual.

In the standard scenarios used for *C. riparius*, we assume an unstructured (homogeneous) aquatic habitat containing only benthic areas with organic substrate (detritus) throughout the entire simulation area (see Figure 5.3.1.1), in which single grid cells can only differ locally in the modelled current food and larval density.

Hence, the individual movement probability between the grid cells is based solely on the effective energy status (scaled reserve density e) of an individual larva, which indirectly reflects the food availability on a grid cell: The hungrier a larva is, the higher the probability of movement.

Once per day, each individual larvae will move to a neighbouring cell if its random number [0..1] for movement, renewed every day for each larvae, exceeds their probability to stay $P_{stay}$[0..1]., with: $P_{stay}$ = scaled reserve density *e* [-].

If the individual decides to move, it randomly selects an adjacent cell within the grid in vertical or horizontal direction.

- **Figure 5.3.1.1: Grid map for an area of 0.5 m² with a standard grid size of 0.025 m² per cell (4 x 5 grid cells) used for ecological scenarios for *Chironomus riparius*.**

# Food resources

For laboratory scenarios, daily food additions at the beginning of each day can be read in from a data file as energy per area [J m^-2^]. All grid cells receive the same amount of food. The supplied food (expressed as food density *X* [J m^-2^] is allowed to accumulate on the sediment surface over time without further degradation and is only reduced by the food consumption of the larvae.

# Feeding

As long as the food density on a specific grid cell is greater than zero, each individual staying on that cell undergoes the feeding process as described below.

The hourly feeding rate ${\dot{\boldsymbol{p}}}_{\boldsymbol{X}}$ of all larvae within one grid cell is calculated in random order based on the maximum daily feeding rate ${\boldsymbol{\{}\dot{\boldsymbol{p}}}_{\boldsymbol{Xm}}\boldsymbol{\}}$, the half-saturation constant $\boldsymbol{K}_{\boldsymbol{S}}$ for food uptake and the temperature dependency *TC*:

$$\dot{p}_{X}=\frac{{\{\dot{p}}_{Xm}\}}{24h}*s_{M}*L^{2}* \frac{X}{K_{S}+X} *TC$$

$\dot{p}_{X}$ : realized hourly feeding (ingestion) rate per individual [J/h]

${\{\dot{p}}_{Xm}\} :$ maximum daily surface-area-specific feeding rate [J/(d * cm^2^)]

$s_{M}$ : acceleration factor [-] $L$ = structural length of individual [cm]

$K_{S}$ : half-saturation constant for food uptake [J/m^2^]

*X* : food density [J m^-2^]

*TC* : temperature correction [-]

$\left\{ \dot{p}_{Xm} \right\}$ is obtained from the value for $\left\{ \dot{p}_{Am} \right\}$ and $\kappa_{X}$, where $\left\{ \dot{p}_{Am} \right\}$assumes the value $\left\{ \dot{p}_{Am} \right\}_{ind}$ when inter-individual variability is taken into account (see Section 5.3.6).

$$\left\{ \dot{p}_{Xm} \right\}= \left\{ \dot{p}_{Am} \right\} / \kappa_{X}$$

$\kappa_{X}$ : fraction of food energy fixed in reserve (assimilation efficiency)

$\left\{ \dot{p}_{Am} \right\}$ : surface-area-specific maximum assimilation rate [J d^-1^ cm^-2^]

The density of food resources *X* in each grid cell is continuously reduced by larval feeding as follows:

Directly after each calculation of the hourly ingestion rate $\dot{p}_{X}$of an individual on a defined grid cell, this value is subtracted from the available food quantity *X* of this grid cell. Within each hour, all larvae on a grid cell always feed in random order.

Over the course of a day (24*h*), the hourly feeding (ingestion) rate per individual is summed up to obtain the individual daily feeding rate $\dot{p}_{X}sum$:

$$\dot{p}_{X}sum= \dot{p}_{X}sum + \dot{p}_{X}$$

$\dot{p}_{X}$sum : realized daily feeding rate per individual [J/d]

$\dot{p}_{X}$: realized hourly feeding rate per individual [J/h]

Based on that daily feeding rate per individual, its realized scaled functional response is calculated with:

$$f:= \dot{p}_{X}sum / ({\{\dot{p}}_{Xm}\}*s_{M}* TC * L^{2})$$

*f* : scaled functional response [-]

$\dot{p}_{X}$sum : daily feeding rate [J/d]

$\dot{p}_{Xm}$ : maximum ingestion rate [J/(d * cm^2^)]

*TC* : temperature correction [-]

*L* : structural length of individual [cm]

Further information related to the used feeding formulas can be found in Section 5.1 (DEB theory).

# Reproduction

Once an individual reached maturity at puperty, the reproduction process is initialised. After pupation and hatching, the semelparous reproduction strategy of *Chironomus riparius* leads to a single reproductive episode of the adult aerial stage before death. In the model, it is assumed that the females are already fertilised and do not need to mate to produce offspring. Each female that has reached sexual maturity produces one clutch of fertilised eggs, from which all larvae are assumed to hatch without further egg mortality.

During reproduction, the brood size per clutch is calculated (see Section 5.1, eq. 12), and the resulting number of offspring per clutch is released.

If offspring is released, the model produces new individuals on the same spatial cell their mother is located and adds these to the population.

# Survival

Death can occur as a result of density dependence mortality, starving, aging.

The starvation and aging processes are part of the DEB calculations and are further described in Section 5.1.

Mortality due to starvation occurs when the individual scaled reserve density is depleted (*e* = 0). No additional mortality due to shrinkage stress was assumed.

*Chironomus riparius* does not die of aging under normal circumstances. With adequate food and typical developmental times, they reach maturity, reproduce, and die after reproduction, before the aging process can influence their lifespan. In cases of insufficient food availability, individuals would die from starvation before aging could become a factor.

The model assumes a fixed, density-dependent mortality rate *hdens* [% d^-1^] that acts continuously over time and depends only on larval density [larvae per area]. Based on density experiments, the daily mortality rate *hdens* can be described by a power function y. The susceptibility of each individual larva to be killed as a result of density-dependent mortality is determined by a uniformly distributed individual random number between 0 and 1, which is renewed daily.

$$h_{dens}={a*density}^{b}$$

Further information of the stochastic aspects related to survival probability, are described in Section 4.6.

# Individual variability of feeding rate in *Chironomus riparius*

In order to reproduce the sigmoidal cumulative emergence pattern in laboratory population level experiments, inter-individual variability of development time was integrated into model. To estimate the individual variability, we followed the approach of Koch and De Schamphelaere (2020), who added the variability to the mean surface-area-specific maximum assimilation rate $\left\{ {\dot{\boldsymbol{p}}}_{\boldsymbol{Am}} \right\}$ of a species. Based on laboratory control data for *C. riparius* emergence without food limitation, a log-normal distribution with a standard deviation 𝜎 of 0.35 delivered the best overall fit (for details, see: Koch et al. (2024), Supplemental Information, S6). When an individual is born, a fixed individual value for $\left\{ {\dot{\boldsymbol{p}}}_{\boldsymbol{Am}} \right\}_{ind}$ is calculated and assigned:

$$f_{pAm}=e^{RandG(\mu, \sigma)}$$

$f_{pAm}$ = individual correction factor sampled at random from the log-normal distribution [-]

RandG = Delphi function for generating random numbers with Gaussian normal distribution

$\mu$ = 0; σ = standard deviation

$\left\{ {\dot{\boldsymbol{p}}}_{\boldsymbol{Am}} \right\}_{ind}$ *=* $\left\{ {\dot{\boldsymbol{p}}}_{\boldsymbol{Am}} \right\}$ *_*_* $f_{pAm}$

$\left\{ {\dot{\boldsymbol{p}}}_{\boldsymbol{Am}} \right\}$: species-specific and surface-area-specific maximum assimilation rate [J d^-1^ cm^-2^]

$\left\{ {\dot{\boldsymbol{p}}}_{\boldsymbol{Am}} \right\}_{ind}$ : individual surface-area-specific maximum assimilation rate [J d^-1^ cm^-2^]

# Calibration routine

The model includes a Downhill-Simplex algorithm to (re-)calibrate selected parameters based on given data sets. The Algorithm was implemented based on the work of (Nelder & Mead 1964, Press et al., 1989), and is designed to find the best parameter combination with a minimum of a function with multiple independent variables.

This search algorithm optimizes the selected parameter set with regard to the error quantifier function F to be minimized, which results from the sum of the squared difference between all measured values m on the observation time points n and the corresponding simulation values s:

F = $\sum_{1}^{n} {(m-s)}^{2}$

Termination of the calibration process occurs when the deviation in the function F between successive calibration runs is below a value of 1E-7. This criterion was usually reached in our scenarios after approx. 250 calibration runs.

# Initialization

For the spatial specification of the environment, a raster map for a hypothetical or real study area is imported as spatially explicit habitat information (txt file) for the model.

For initialisation of the population, individual parameters, the starting density of the larvae and their intended length distribution of the starting population must be defined.

Simulations are initiated by setting the initial starting number as individuals per m². The scenario-specific initial larval density [larvae m^-2^] is achieved by a random distribution of the required larvae to all cells of the grid map.

The individual correction factor $f_{pAm}$ of the maximum assimilation rate $\dot{p}_{Am}$ [J/d] is sampled for each larva from a log-normal distribution as described in chapter 5.3.6.

During the initialization process the DEB state parameters of the individuals are calculated based on the DEB rules. Throughout this process, we assume no energy limitation for the individuals and the process ends with achieving the starting size of the individuals within the desired population size distribution is reached.

To initialise the size distribution of the population, either a fixed value for the body lengths or a range (with specification of mean value and standard deviation for a normal distribution) between minimum and maximum body lengths can be specified. The body length is converted into a maturity level within the model.

The length [cm] of each individual is drawn from a normal distribution of the intended population structure at simulation start. Within the possible length range of the young larvae (between ‘maturity at birth’ and ‘maturity at puberty’), an intended length distribution can be defined for the start of the simulation. For each starting individual, a length is randomly selected from a normal distribution based on the specified mean value M and standard deviation SD, taking into account the permissible minimum and maximum lengths.

To generate only larvae freshly hatched from the egg, the length at birth can be chosen (with SD=0), and the DEB state parameters will be set according to the parameter values for 'maturity at birth' (Koch et al. 2024).

The initialization routine is completed as soon as all individuals have reached their intended initial size.

The starting conditions must be initiated for food availability in laboratory scenarios (feeding scenarios).

For laboratory conditions, the initial food initial energy density of all grid cells was set to ${ini}_{X}$ [J/m²] depending on the experimental conditions at the test start. The parameter values for the initial setting for the *C. riparius* laboratory study can be found in Section 8.

# Case study-specific parameters for *C. riparius*

This section contains the physiological and environmental parameters of the Chironomus IBM population model.

The module for *Chironomus riparius* was parameterized in detail using laboratory experiments at different temperatures (Koch et al., 2024) to calibrate the physiological DEB-TKTD model for *C. riparius* based on dynamic energy budget (DEB) theory, as well as further experiments on density dependence at different food and larval densities (Strauss et al., 2025).

Table 8.1: Physiological DEB parameters of the hax type model for *Chironomus riparius* used in this study (Koch et al., 2024)*

| **Symbol** | **Unit** | **Interpretation** | **Value** |
| --- | --- | --- | --- |
| ${\{\dot{F}}_{m}\}$ | L d^−1^ cm^−2^ | Specific searching rate | 6.5 (fixed) |
| $\kappa_{X}$ | − | Assimilation efficiency | 0.8 (fixed) |
| ${\{\dot{p}}_{Am}\}$ | J d^−1^ cm^−2^ | Surface-area-specific maximum assimilation rate | 816.20 |
| $\dot{v}$ | cm d^−1^ | Energy conductance | 0.0154 |
| $\kappa$ | − | Allocation fraction to soma | 0.400 |
| $\kappa_{R}$ | − | Reproduction efficiency | 0.95 (fixed) |
| ${[\dot{p}}_{M}]$ | J d^−1^ cm^−3^ | Volume-specific somatic maintenance rate | 3474 |
| ${\{\dot{p}}_{T}\}$ | J d^−1^ cm^−2^ | Surface-area-specific somatic maintenance rate | 0 (fixed) |
| $\dot{k}_{J}$ | d^−1^ | Maturity maintenance rate coefficient | 0.002 (fixed) |
| $[E_{G}]$ | J cm^−3^ | Volume-specific costs for structure | 4400 (fixed) |
| $E_{H}^{b}$ | J | Maturity level at birth | 5.31 × 10^-3^ |
| $E_{H}^{p}$ | J | Maturity level at puberty | 1.74 × 10^-2^ |
| $E_{H}^{e}$ | J | Maturity level at emergence | 3.79 × 10^-3^ |
| ${[E_{R}]}_{j}$ | J cm^−3^ | Reproduction buffer density at pupation (independent of $f$ in the default hax model but assumed to be only true at $f=1$ in this study) | 3.54 × 10^4^ |
| ${{(E}_{R}/L)}_{j}$ | J cm^−1^ | Reproduction-buffer-to-structural-length ratio at pupation (assumed to be independent of $f$ in this study) | 195.08 |
| $\kappa_{V}$ | − | Energy conversion efficiency from reserve to structure (during growth) and back to reserve (at pupation) | 0.7 (fixed) |
| $T_{REF}$ | K | Reference temperature | 293.15 (20°C, fixed) |
| $T_{A}$ | K | Arrhenius temperature | 7377 |
| $T_{H}$ | K | Upper boundary temperature | 293.6 |
| $T_{AH}$ | K | Arrhenius temperature for upper boundary | 5949 |

*: The basic DEB model was calibrated using laboratory chironomid studies (see Koch et al. 2024).

Table 8.3: Additional population and environmental parameter

| **Symbol** | **Unit** | **Interpretation** | **Value** | **Source** |
| --- | --- | --- | --- | --- |
| ***Chironomus riparius* life history parameters** | | | |  |
| $a$ | − | Factor of density dependent mortality h*dens* | 0.29 for larval density per 50 cm^2^, corresponding to a value of 0.035 for larval density per m^2^ | Calibrated (on lab density experiments, Strauss et al. 2025) |
| $b$ | − | Coefficient of density dependent mortality h*dens* | 0.4 | Measured (from lab density experiments, Strauss et al. 2025) |
| $K$*_s_* | J/m² | half saturation constant for food uptake | 1045 | Calibrated (on lab density experiments, Strauss et al. 2025) |
| Length_b_ | cm | Larval length at birth | 0.142 | Koch et al. (2024) |
| **Environmental parameters** | | | |  |
| ${ini}_{X}$ | J/m² | Initial food density | 0 | Experimental setting |

# Data input

The IBM Chironomus population model was implemented in Delphi^®^ Embarcadero 2010 RAD studio^®^ XE2.

Technically, the model requires five data files as input. These files contain the species and habitat parameters, water temperature profiles, etc. (see Table 9.1).

Table 9.1: Overview input files

| Input files | Content |
| --- | --- |
| spec.xml | - DEB parameter of the modelled species - Additional life-cycle parameters of the modelled species - Specification of the intended size distribution of the starting population |
| settings.txt | - Simulation settings  (e.g., initial values, food resource parameters, simulation time, output settings) |
| temp.txt | - Temperature profile (daily time step) |
| site_information.txt | - General properties of the site (waterbody) - Grid map of habitat properties |
| Biomass.txt | - Initial number of larvae - Additional parameters:  - half saturation constant K_s_ for food uptake - factor *a* for the density dependent mortality |

# References

Álvarez, O.A., Jager, T., Redondo, E.M., Kammenga, J.E., 2006. Physiological modes of action of toxic chemicals in the nematode Acrobeloides nanus. Environmental Toxicology and Chemistry 25, 3230–3237. https://doi.org/10.1897/06-097R.1

Augustine S, Gergs A. (2019): AmP Chironomus riparius, Version 2019/09/21. Available from: https://www.bio.vu.nl/thb/deb/deblab/add_my_pet/entries_web/Chironomus_riparius/Chironomus_ripariu s_res.html

Forsythe, W.C., Rykiel Jr, E.J., Stahl, R.S., Wu, H., Schoolfield, R.M. (1995): A model comparison for daylength as a function of latitude and day of year. Ecological Modelling 80, 87–95.

Grimm, V., Railsback, S.F., Vincenot, C.E., Berger, U., Gallagher, C., DeAngelis, D.L., Edmonds, B., Ge, J., Giske, J., Groeneveld, J., Johnston, A.S.A., Milles, A., Nabe-Nielsen, J., Polhill, J.G., Radchuk, V., Rohwäder, M.-S., Stillman, R.A., Thiele, J.C., Ayllón, D., 2020. The ODD Protocol for Describing Agent-Based and Other Simulation Models: A Second Update to Improve Clarity, Replication, and Structural Realism. Journal of Artificial Societies and Social Simulation 23. https://doi.org/10.18564/jasss.4259

Hendriks, A.J., 1999. Allometric Scaling of Rate, Age and Density Parameters in Ecological Models. Oikos 86, 293–310. https://doi.org/10.2307/3546447

Holling, C.S., 1959. The Components of Predation as Revealed by a Study of Small-Mammal Predation of the European Pine Sawfly. The Canadian Entomologist 91, 293–320. https://doi.org/10.4039/Ent91293-5

Jager, T., Zimmer, E.I., 2012. Simplified Dynamic Energy Budget model for analysing ecotoxicity data. Ecological Modelling 225, 74–81. https://doi.org/10.1016/j.ecolmodel.2011.11.012

Jeschke, J.M., Kopp, M., Tollrian, R., 2002. Predator Functional Responses: Discriminating Between Handling and Digesting Prey. Ecological Monographs 72, 95–112. https://doi.org/10.1890/0012-9615(2002)072[0095:PFRDBH]2.0.CO;2

Koch, J., De Schamphelaere, K. A., 2020. Estimating inter-individual variability of dynamic energy budget model parameters for the copepod *Nitocra spinipes* from existing life-history data. Ecological Modelling, 431, 109091.

Koch, J., Classen, S., Gerth, D., Dallmann, N., Strauss, T., Vaugeois, M., Galic, N., 2024. Modeling temperature-dependent life-cycle toxicity of thiamethoxam in *Chironomus riparius* using a DEB-TKTD model. Ecotoxicology and Environmental Safety 277, 116355. https://doi.org/10.1016/j.ecoenv.2024.116355

Kooijman, S.A.L.M., 2014. Metabolic acceleration in animal ontogeny: An evolutionary perspective. Journal of Sea Research, Dynamic Energy Budget theory: applications in marine sciences and fishery biology 94, 128–137. https://doi.org/10.1016/j.seares.2014.06.005

Kooijman, S.A.L.M., 2010. Dynamic energy budget theory for metabolic organisation, 3. ed. Cambridge Univ. Press, Cambridge.

Nelder JA, Mead R (1964): A simplex method for function minimization. Comput. J. 7: 308-313.

Press, W.H., Press, W.H., Flannery, B.P., Teukolsky, S.A., Vetterling, W.T., Flannery, B.P., Vetterling, W.T., 1989. Numerical Recipes in Pascal (First Edition): The Art of Scientific Computing. Cambridge University Press.

Rakel, K., Becker, D., Bussen, D., Classen, S., Preuss, T., Strauss, T., Zenker, A., Gergs, A., 2022. Physiological Dependency Explains Temperature Differences in Sensitivity Towards Chemical Exposure. Arch Environ Contam Toxicol 83, 349–360. https://doi.org/10.1007/s00244-022-00963-2

Strauss, T., Gerhard, J., Gerth, D., Koch, J., Ottermanns, R., Vaugeois, M., Galic, N. (2025): What is regulating chironomid populations? The influence of food supply and interference competition on development and mortality in Chironomus riparius. Ecology and Evolution DOI:10.1002/ece3.71949

van der Meer, J., 2006. An introduction to Dynamic Energy Budget (DEB) models with special emphasis on parameter estimation. Journal of Sea Research 56, 85–102. https://doi.org/10.1016/j.seares.2006.03.001

Zimmer, E.I., Ducrot, V., Jager, T., Koene, J., Lagadic, L., Kooijman, S.A.L.M., 2014. Metabolic acceleration in the pond snail *Lymnaea stagnalis*? Journal of Sea Research 94, 84–91. https://doi.org/10.1016/j.seares.2014.07.006
